# An undiscovered circadian clock to regulate phytoplankton photosynthesis

**DOI:** 10.1093/pnasnexus/pgae497

**Published:** 2024-11-06

**Authors:** Yixi Su, Jingyan Hu, Mengsheng Xia, Jiwei Chen, Weizhao Meng, Cheng Qian, Yuexuan Shu, Chao Wang, Xianwei Wang, Kourosh Salehi-Ashtiani, Sigurður Brynjólfsson, Jianping Lin, Yongquan Li, Haisheng Zhang, Lizhong Wang, Weiqi Fu

**Affiliations:** Ocean College, Zhejiang University, Zhoushan 316021, Zhejiang, China; Ocean Research Center of Zhoushan, Zhejiang University, Zhoushan 316021, China; Center for Systems Biology and Faculty of Industrial Engineering, School of Engineering and Natural Sciences, University of Iceland, Reykjavík 101, Iceland; Ocean College, Zhejiang University, Zhoushan 316021, Zhejiang, China; Ocean College, Zhejiang University, Zhoushan 316021, Zhejiang, China; Ocean College, Zhejiang University, Zhoushan 316021, Zhejiang, China; Ocean College, Zhejiang University, Zhoushan 316021, Zhejiang, China; Ocean College, Zhejiang University, Zhoushan 316021, Zhejiang, China; Ocean College, Zhejiang University, Zhoushan 316021, Zhejiang, China; Ocean College, Zhejiang University, Zhoushan 316021, Zhejiang, China; School of Oceanography, Shanghai Jiao Tong University, Shanghai 200030, China; Laboratory of Algal, Systems, and Synthetic Biology, Division of Science and Math & Center for Genomics and Systems Biology, New York University Abu Dhabi, Abu Dhabi 129188, UAE; Center for Systems Biology and Faculty of Industrial Engineering, School of Engineering and Natural Sciences, University of Iceland, Reykjavík 101, Iceland; Key Laboratory of Biomass Chemical Engineering of Ministry of Education, College of Chemical and Biological Engineering, Zhejiang University, Hangzhou 310058, Zhejiang, China; Institute of Pharmaceutical Biotechnology & Research Center for Clinical Pharmacy, The First Affiliated Hospital, School of Medicine, Zhejiang University, Hangzhou 310058, Zhejiang, China; Ocean College, Zhejiang University, Zhoushan 316021, Zhejiang, China; Ocean Academy, Zhejiang University, Zhoushan 316021, Zhejiang, China; Ocean College, Zhejiang University, Zhoushan 316021, Zhejiang, China; Key Laboratory of Offshore Geotechnics and Material of Zhejiang Province, College of Civil Engineering and Architecture, Zhejiang University, Hangzhou 310058, Zhejiang, China; Ocean College, Zhejiang University, Zhoushan 316021, Zhejiang, China; Ocean Research Center of Zhoushan, Zhejiang University, Zhoushan 316021, China; Center for Systems Biology and Faculty of Industrial Engineering, School of Engineering and Natural Sciences, University of Iceland, Reykjavík 101, Iceland; Donghai Laboratory, Zhoushan 316021, Zhejiang, China

**Keywords:** photosynthesis, circadian clocks, phytoplankton, intrinsic circadian rhythm, *Phaeodactylum tricornutum*

## Abstract

Circadian clocks exist in all types of organisms and coordinate key biological processes, e.g. photosynthesis in phytoplankton (microalgae) and land plants. We asked whether a circadian rhythm sustains in phytoplankton when living under constant illumination without environmental cues. Here, we report the first transcriptomic architecture of persistent oscillatory gene expression in the model marine diatom, *Phaeodactylum tricornutum* living under constant illumination and temperature without environmental cues. We show that cyclic expression of a considerable number of genes involved in light harvesting and carbon fixation sustained after 24 h of constant illumination (free-running), which could pose additional constraints on cell growth under constant light conditions. Over long-term adaptation to constant illumination, the majority of the rhythmic genes identified under diel light conditions lose their oscillatory expression in the absence of external entrainers, and the genes potentially controlled by persistent circadian clocks are primarily involved in transcriptional regulation and cell division. We find constant illumination leads to an increased average expression of transcription factors and cell division genes, while genes involved in the Calvin–Benson cycle and pigment biosynthesis are kept at low expression levels, which plays a role in the down-regulation of photosynthetic efficiency. By manipulation of the dark rest period, we confirm a fine-tuned light/dark cycle could dramatically improve photosynthetic efficiency in microalgae. Our results unveil a novel persistent circadian rhythm on photosynthetic regulation in marine phytoplankton and provide critical insights into the interaction between environmental signals and inheritable internal circadian clocks in diatoms.

**Significance Statement**

Phytoplankton (microalgae) accounts for approximately half of the global photosynthetic activity and primary production. Further exploration of microalgae for biotechnological platforms will rely in large part on improved photosynthesis. Here, we show evidence that a circadian rhythm identified under constant conditions without environmental cues results in photosynthetic inefficiency in diatoms, indicating an undiscovered circadian clock exists with more robustness than previously reported. We unveil the self-sustained circadian clock drives the down-regulation of pigment biosynthesis and carbon fixation, limiting the efficiency of prolonged photosynthesis. We further show that a short-term dark rest dramatically improves photosynthetic efficiency in microalgae by manipulation of the light/dark cycle, shedding light on fundamental features of photobiology, and advancing the understanding of circadian rhythms in photosynthesis regulation.

## Introduction

Our planet Earth is home to billions of different species of organisms. Biological rhythms (clocks) are ubiquitous across almost all living organisms, as a consequence of adaptation to fluctuating environments (1, 2). Various rhythmic behaviors of life are shaped by daily and seasonal changes caused by the Earth's rotation and revolution. Among these rhythms, circadian clocks, which follow a ∼24-h cycle synchronized with solar time, have profound biochemical and molecular effects on life (3, 4). These biological clocks can be reset by environmental cues; for example, “jet lag” occurs immediately after crossing time zones and is alleviated as one adapts to the local time zones. Unlike human beings, photosynthetic organisms such as land plants and phytoplankton (microalgae) rely on circadian clocks to optimize their photosynthesis in response to daily light/dark (LD) cycles (5–7). Phytoplankton, in particular, accounts for over half of the global primary production via photosynthesis and plays a crucial role in sustaining ecosystems and supporting the economy (3, 8–11). With the global push toward carbon neutrality, understanding the circadian rhythms of photosynthetic organisms has become critical to enhancing their efficiency in carbon fixation. It is estimated that increasing photosynthetic efficiency by 10% could additionally capture 40 billion tons of carbon dioxide (CO₂) annually, a quantity comparable to the CO₂ emissions from fossil fuels in 2021 (12, 13). Therefore, optimizing photosynthesis through a deeper understanding of the circadian mechanisms in phytoplankton could revolutionize sustainable carbon capture technologies.

**Fig. 1.**
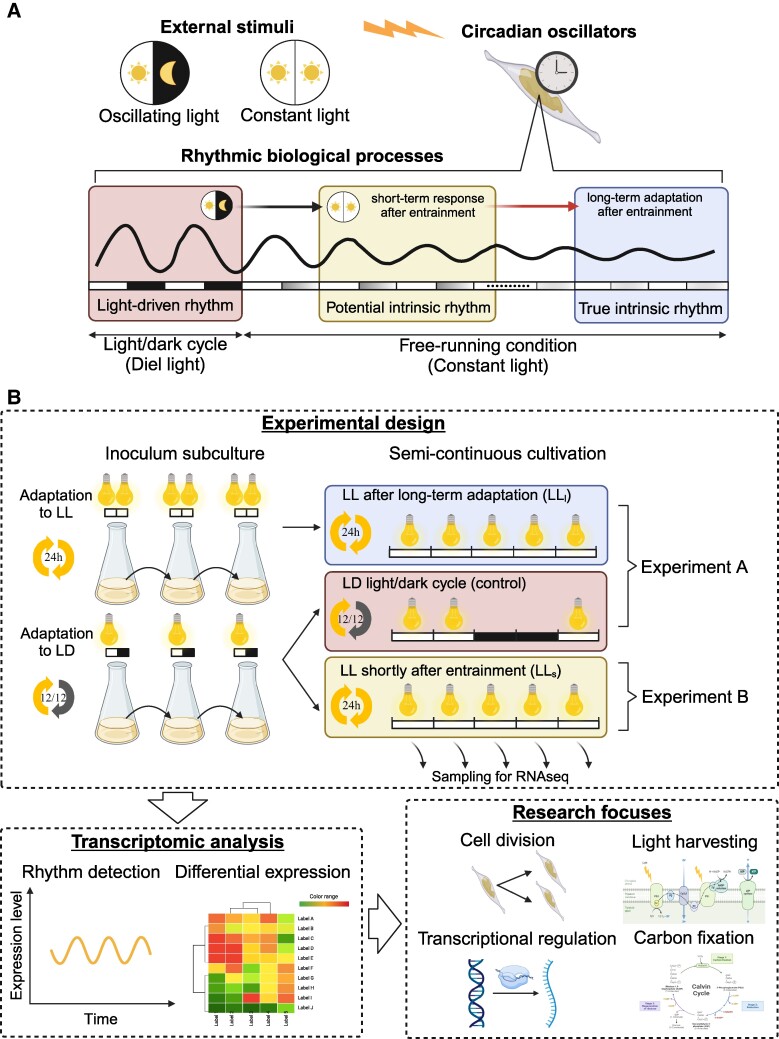
Schematic representation of the circadian system and experimental design. A) Light-driven rhythm and intrinsic rhythm. B) Experimental design and procedures. Semi-continuous cultivations were conducted under controlled conditions with fixed temperature, light intensity, and pH. Cell density and culture volumes were maintained by adding fresh media and removing cultures under a defined time interval. LL, constant light; LD, 12 h/12 h LD cycle; LL_l_, long-term adaptation to constant light conditions after entrainment; LLs, constant light conditions shortly after entrainment.

One challenge in microalgal production is the compromised photosynthetic efficiency under continuous illumination. While longer photoperiods are expected to boost biomass production, the reality turns out to be the opposite, implying impaired photosynthesis due to overly sustained light exposure (14, 15). Oscillatory growths, physiological states, and cellular compositions of microalgae have been observed under constant culturing conditions (16, 17) as shown in Fig. 1A. In nature, peak primary production in the Arctic Ocean (18) and the Southern Ocean typically occurs away from the summer solstice, despite the extended daylight hours during this period (e.g. polar day with 24-h daylight) (19). Specifically, the primary production in the Weddell Sea sector of the Southern Ocean peaks in January, equivalent to July in the Northern Hemisphere (20). These phenomena hint at an internal circadian clock that continues to function even under constant light (LL) conditions, which may be responsible for the reduced photosynthetic efficiency (21, 22). Despite extensive studies on diel transcriptome changes in microalgae under LD cycles (7, 23–27), the global transcriptomic rhythms in phytoplankton under continuous illumination are yet to be explored. Furthermore, it remains unknown whether rhythms persist in phytoplankton under prolonged constant light and temperature conditions (free-running conditions).

This study addresses this gap by examining the circadian transcriptomes of diatoms, a dominant group of phytoplankton, under constant light and temperature conditions. Unlike previous studies that focused on the periodic expression of specific genes using microarray or quantitative PCR analysis (28–30), this study marks the first investigation of global transcriptomic rhythms in diatoms. By looking into the diatom cultures after their adaptation to constant conditions, we aim to determine whether robust circadian rhythms persist and to decipher the molecular basis of undiscovered circadian regulation in phytoplankton. This study provides a new perspective to enhance the regulation of photosynthetic processes, both in natural ecosystems and in the design of photosynthetic cell factories for sustainable development.

## Results and discussion

### Physiological status of *P. tricornutum* throughout semi-continuous cultivations

To reveal external stimuli-independent rhythms in the model diatom *P. tricornutum*, this study compared parallel cultivations under LL and LD conditions (Fig. 1B). Prior to experiments, the inoculum culture was incubated under LL for more than six months with regular subculturing to eliminate any light-responsive rhythms inherited from a day/night cycle (Fig. 1B). After adapting to a 12:12 LD cycle for two weeks, the LD group exhibited diel oscillations in growth rate and quantum yields (SI Appendix, Fig. S2), as previously reported (7, 17, 31). Even in nonentrained cultures, periodic growth rate and chlorophyll fluorescence could be observed under the LL condition during batch cultivation (SI Appendix, Fig. S1).

In *P. tricornutum*, circadian rhythms in growth rate, cell size, and pigment contents remained for a short period after switching from the LD cycle to LL conditions (32). Additionally, oscillatory chlorophyll fluorescence was observed in *P. tricornutum* under LL conditions for up to four days following entrainment to the LD cycle (28). For an extended period, the oscillations in the physiological parameters became dampened, likely due to the desynchronization of the cell division cycle (32). It is believed that, under constant light, cell activities in the culture become randomized as the daily resetting of the diel rhythm is abolished (17, 30, 32). In a desynchronized culture, measurements from a bulk culture tend to average out, leading to flat trends even if intrinsic rhythms exist at the single-cell level. However, periodicities in growth rate and quantum yield of nonentrained cultures were reported for the first time in this study and confirmed by repeated experiments. A possible explanation is that the proliferating cells, derived from a diluted population, might have been in synchrony to a certain extent. Due to the decreasing trend in photosynthetic efficiency as cell density increased, periodic growth rates and quantum yield were only observable within two days after inoculation (SI Appendix, Fig. S1).

Since rhythms in batch cultures could not be sustained, semi-continuous cultivations were conducted under constant light intensity, temperature, and pH, along with well-controlled cell density and nutrient levels, to eliminate external stimuli other than photoperiod. Consistent with previous studies (31, 32), biomass productivity in cultures exposed to continuous illumination (i.e. 24 h per day) did not result in twice the productivity compared with the LD (12:12) cycle (SI Appendix, Table S1), suggesting a lower biomass yield under the LL condition. However, regular rhythms could not be observed during the semi-continuous cultivation under constant light (SI Appendix, Fig. S2). This may be due to fluctuations caused by the addition of fresh media, which could have masked the rhythms that were otherwise observable in batch cultures.

### The periodic gene expression under constant and diel illumination

Previous studies on circadian rhythms only investigated microalgal cultures immediately after entrainment through LD cycles, which could interfere with an overlap of residual regulation from the LD conditions. To address this issue, we used LL-adapted cultures without prior LD entrainment in this study to investigate the intrinsic rhythms of microalgae (SI Appendix, Table S2). Time-series transcriptome data, covering 24 h during the semi-continuous cultivation. For comparison, LD-entrained cultures and LL-adapted cultures were cultivated under LD and LL conditions, respectively, in Experiment A. Due to the low sampling frequency, which made it challenging to discern fine-scale phase differences, we did not aim to investigate phase shifts under constant light conditions. Instead, the analysis focused on a period of 24 h to identify genes with potentially cyclic expression patterns. In addition, Experiment B was conducted to supplement Experiment A by studying the short-term response of LD-entrained culture to the LL conditions. As a result, this study generated datasets under three conditions: (ⅰ) LD-entrained culture grown under LD cycle conditions as the control, (ⅱ) LD-entrained culture grown under LL conditions representing short-term (LL_s_) free-running conditions, and (ⅲ) LL-adapted culture grown under LL conditions representing long-term (LL_l_) free-running conditions.

In total, 10,849 transcripts with an average fragment per kilobase exon per million mapped reads (FPKM) > 1 were identified in the LD group, while the numbers of transcripts in the LL_s_ and LL_l_ groups were 10,666 and 10,741, respectively. Rhythmicity analysis using the Jonckheere–Terpstra–Kendall (JTK) test revealed that 7,343 genes were responsive to diel light conditions in the LD group. Under constant light conditions, 5,245 and 1,377 genes were identified as rhythmic genes in the LL_s_ and LL_l_ groups, respectively, showing significant cyclic expression (*P*_adj_ < 0.05). These datasets were further analyzed using RAIN (33), an alternative nonparametric method for rhythm detection, which yielded results highly consistent with JTK, showing at least 96.7% overlap in identified genes (SI Appendix, Fig. S3). RAIN identified a higher number of rhythmic genes, as it could detect additional asymmetric sawtooth-shaped waveforms that are undetectable by the JTK algorithm (33). The identified rhythmic genes were then screened to exclude those with low Max/Min fold change (maximum FPKM/minimum FPKM < 1.3) and low amplitude (amp < 1). As a result, the number of rhythmic genes potentially contributing to significant oscillatory outputs was 6,978 in the LD group, representing 64.3% of the transcribed protein-coding genes of *P. tricornutum* under the LD condition. This was comparable to previous transcriptomic studies on *P. tricornutum*, which identified 5,803 (26) and 4,567 (7) rhythmic genes.

Through the screening, the LL_s_ group exhibited 4,277 rhythmic genes on the second day (24 to 48 h of constant illumination) after LD entrainment. An extended dataset covering 48 h was also analyzed, resulting in 2,564 rhythmic genes, of which 1,970 genes are consistent with those identified in the 24-h data (SI Appendix, Fig. S4). The additional 24 h of data led to fewer number of rhythmic genes. Apart from more stringent detection across two cyclic periods, the elimination of LD regulation and desynchronization under constant illumination could contribute to this reduction. Notably, the number of rhythmic genes identified using the third day (48 to 72 h of constant illumination) data sharply decreased to 1,956 genes. Additionally, setting a flexible period between 20 and 28 h with the 48-h dataset revealed 777, 1,390, and 863 genes with cyclic patterns following 20, 24, and 28-h periods, respectively. A further comparison of these genes with those identified using fixed 24-h periods demonstrated that most of the rhythmic genes detected in flexible periods (24 ± 4 h) were also identified by the fixed period using either the 24-h dataset or the 48-h dataset (SI Appendix, Fig. S4).

In the nonentrained culture, only 1,028 rhythmic genes remained under constant light, accounting for 9.6% of all detected transcripts. The ratio of amplitude to maximum FPKM was calculated for each cyclic gene to evaluate the relative change of oscillation. Most LD-derived rhythmic genes (81.0%) showed relative amplitudes in a range of 10–40% (SI Appendix, Fig. S5). In contrast, the amplitude of the majority of LL-rhythmic genes was concentrated within a narrower range of 10–20% of the maximum expression level, indicating a relatively stable expression of the genes under constant light conditions. These findings suggest that the diel expression of cyclic genes largely depends on external light stimuli. In the absence of entrainment, a significant number of genes still exhibited cyclic expression, albeit with weak oscillations. However, over the long term in free-running conditions, the diatom *P. tricornutum* lost most of its rhythmicity at the transcriptomic level, reflecting the arrhythmic physiological parameters. Nonetheless, a small proportion of genes persisted in circadian rhythms independent of environmental cues. To the best of our knowledge, the present study provides the first evidence of the free-running circadian transcriptome under constant conditions in diatoms.

**Fig. 2.**
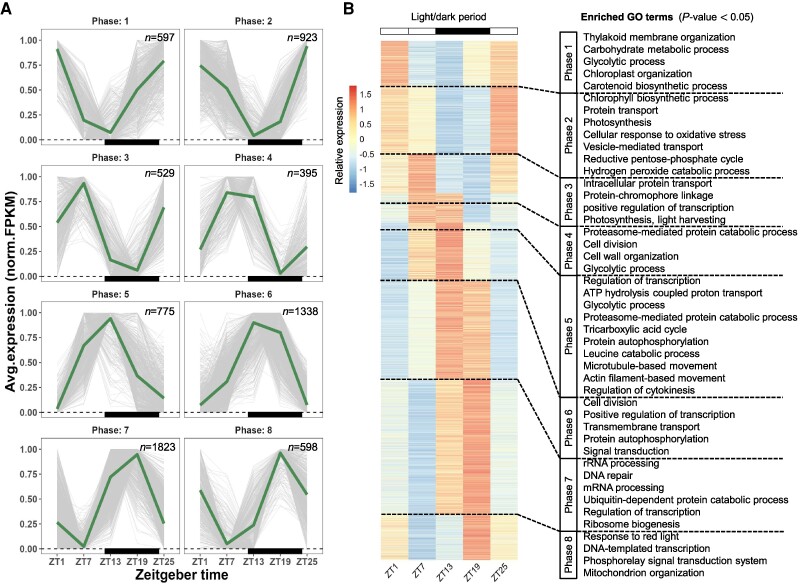
Clustering of rhythmic genes under LD cycle conditions. A) Trendline of 24 h periodic change in transcript expression of rhythmic genes. FPKM values were normalized between 0 and 1; the thick lines represent an averaged expression of each cluster in the 12 h/12 h LD cycle conditions; “*n*” represents the number of genes in each cluster. Black squares indicate dark periods. B) Heatmap of gene clusters associated with enriched GO terms.

**Fig. 3.**
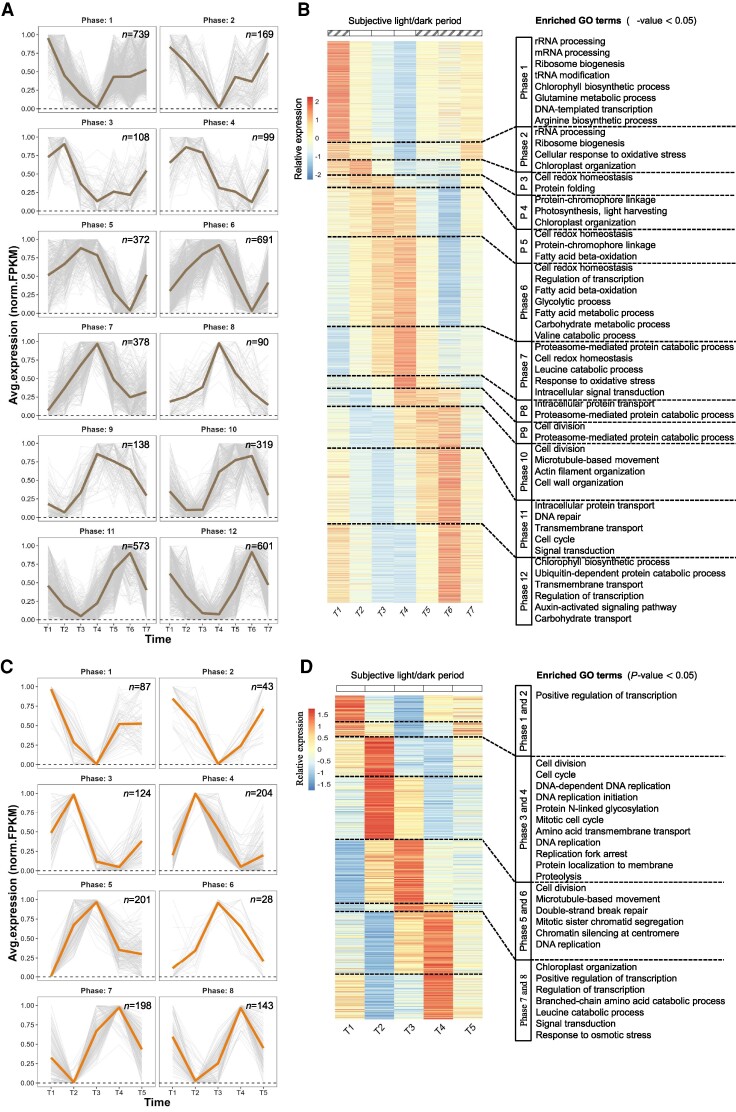
Clustering of rhythmic genes in entrained and nonentrained culture under constant light conditions (LL). A) and C) Trendline of 24-h periodic change in transcript expression of rhythmic genes. FPKM values were normalized between 0 and 1; the thick lines represent an averaged expression of each cluster in short-term LL condition (LL_s_) and long-term LL condition (LL_l_), respectively; “*n*” represents the number of genes in each cluster. Striped squares indicate subjective dark periods. B) and D) Heatmap of gene clusters associated with enriched GO terms.

Depending on the sampling frequency (6 h in Experiment A, 4 h in Experiment B), the identified rhythmic genes were clustered into either eight or 12 phases based on their expression patterns (Figs. 2 and 3). The distribution of the expression phases in the LD group exhibited a bimodal pattern, with relatively more genes peaking in the morning (phase 2) and midnight (phases 6 and 7) (SI Appendix, Fig. S5B). Similar observations have been reported in other species, such as *Nannochloroplsis oceanica* (17), *Seminavis robusta* (24), *Chlamydomonas reinhardtii*, *Porphyridium purpureum*, and *Synechocystis* sp. PCC6803 (25). Furthermore, a clear bimodal pattern could be observed after constant illumination for 24 h, but the absence of entrainment shifted the peaking phases of rhythmic genes (SI Appendix, Fig. S5D). In contrast, due to the lack of external stimuli to entrain and synchronize gene expression into coherent patterns, rhythmic genes in the LL_s_ group displayed a more scattered pattern of peaking phases (SI Appendix, Fig. S5F).

### Rhythmic patterns associated with specific biological processes

Enrichment analysis of Gene Ontology (GO) annotation was performed to characterize the main functional activities within each phase group. Rhythmic genes in each group were clustered based on gene expression patterns, allowing functional specialization to the enriched GO terms.

In the LD regime, our data align with previous transcriptomic studies on *P. tricornutum* (7, 26), particularly regarding the rhythmic order of active biological processes (Fig. 2). For instance, we observed a predominant up-regulation of photosynthesis, the Calvin–Benson cycle, carbohydrate metabolism during the day, and an elevated expression of glycolysis and the tricarboxylic acid cycle in the dark period, which is consistent with previous findings (7, 26). Processes involved in cell division were activated from phases 4 to 6, in line with reports of active cell division at the transition from light to dark in diatoms and green microalgae (7, 24, 27). Additionally, we found that the proteosome-mediated protein degradation became active several hours before the dark period, while terms such as rRNA processing and ribosome biogenesis predominated in the phase several hours before light onset. These findings suggest that protein degradation and synthesis were separately activated during the day and night, respectively, as previously reported in other microalgal species (17, 34).

The LL_s_ group represents a 24-h dataset of transcriptomes collected after 24 h of illumination following LD entrainment (Fig. 3A). The phases observed in the diel rhythm were largely conserved during subjective day and night periods (Fig. 3B). Genes involved in DNA repair, rRNA/mRNA processing, ribosome biogenesis, signal transduction, and ubiquitin-dependent protein catabolic process were typically activated during the subjective dark period. GO terms related to the cell cycle, such as cell division, microtubule/actin filament-based movement, cell wall organization, and proteosome-mediated protein catabolic process, were enriched in phases spanning the subjective light-to-dark transition. However, in the absence of external stimuli, the timing of some functional activations differed from those in the LD group. For instance, the peaks of gene expression involved in the glycolytic process and leucine catabolic process, which occurred during the dark period of the LD cycle, shifted to the subjective light phase before transitioning into the subjective dark periods in the LL_s_ group. Notably, there was a significantly decreased in the number of rhythmic genes clustered in the early phases of the subjective light period (phases 2 to 5) compared with the corresponding light period (phases 1 to 3) in the LD group. These results indicate that many genes related to photosynthesis and chlorophyll biosynthesis lost their periodic expression under constant light conditions.

To minimize effects of the small cluster sizes in the LL_l_ group, genes from phases with similar trend lines were combined for the enrichment analysis (Fig. 3C). Notably, most of the enriched functions among LL_l_-rhythmic genes were associated with cell division, including various GO terms relevant to chromatin organization, DNA replication, and mitosis. The expression clusters spanning phases 3 to 6 included more than half of the total identified rhythmic genes in the LL_l_ group. Specifically, the combined phases 3 and 4, which possessed more genes encoding G2 mitotic-specific cyclins, minichromosome maintenance complex-binding proteins (MCM), as well as DNA polymerases and DNA mismatch repair proteins, were presumably related to the interphase (G1-S-G2), characterized by DNA synthesis and replication. The subsequent phases 5 and 6 exhibited a peak in the expression of multiple genes encoding structural maintenance of chromosomes proteins, cohesion complex subunits, and microtubule components such as kinesin-like protein, and kinetochores, all of which are involved in the mitotic process (35). These results demonstrated an intrinsic regulation of cell division genes, largely independent of external stimuli.

### Rhythms in global regulation and cell growth

**Fig. 4.**
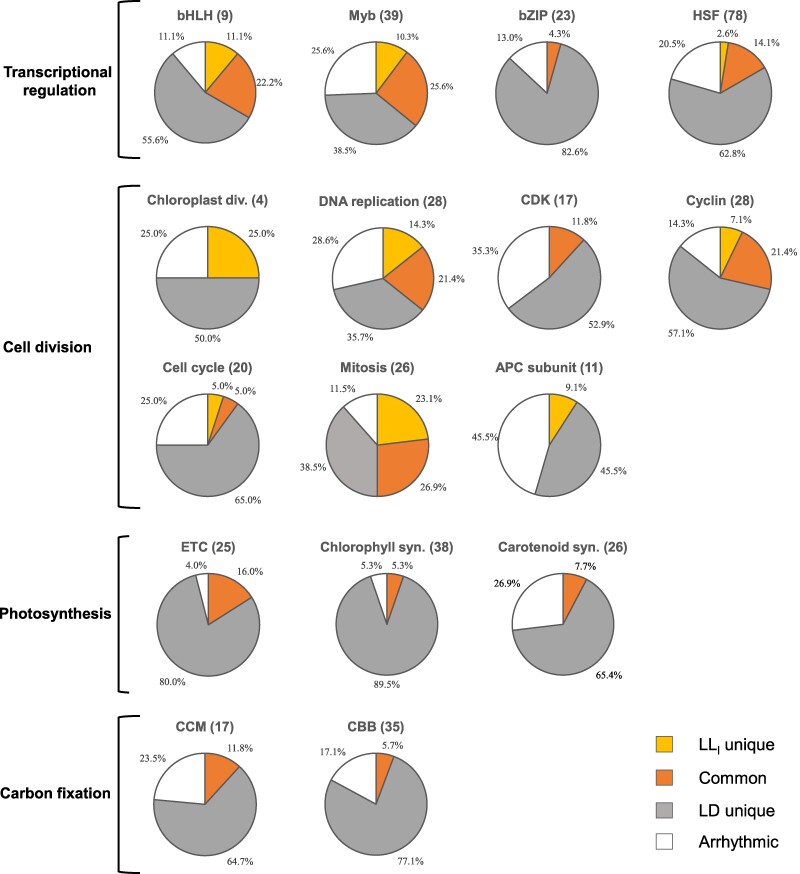

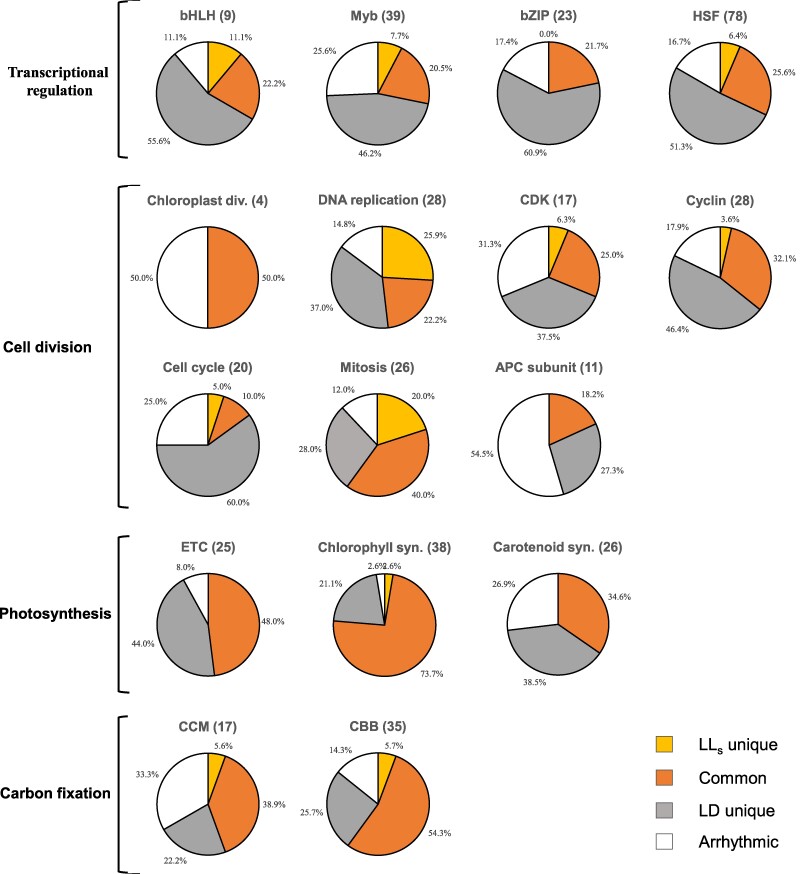
Proportions of self-sustained rhythmic genes in selected biological processes in the long-term constant light (LL_l_) group compared with the LD cycle group. For a specific function or pathway, all annotated genes present in *P. tricornutum* were searched on PLAZA (https://bioinformatics.psb.ugent.be/plaza/versions/plaza_diatoms_01/). The pie charts indicate the proportions of LL_l_- or LD-derived rhythmic genes within each selected functional group. bHLH, basic-helix-loop-helix; Myb; bZIP, basic region leucine zipper; HSF, heat shock factor; Chloroplast div., chloroplast division; Chlorophyll syn., chlorophyll synthesis; Carotenoid syn., carotenoid synthesis; CCM, carbon concentrating mechanism; CBB, Calvin–Benson–Bassham cycle; CDK, cyclin-dependent kinase; APC, anaphase-promoting complex; ETC, electron transport chain.

Then, we investigated the involvement of the identified rhythmic genes in transcriptional regulation and cell growth-related processes, specifically focusing on cell division, photosynthesis, and carbon fixation (Fig. 4). To gather comprehensive information about genes and their functions, all annotated genes associated with the relevant processes of interest were searched in the PLAZA database. The specific genes and their corresponding annotations are available in SI Appendix, Dataset S7.

In this analysis, more than three-fourths of the examined transcription factors (TFs) exhibited rhythmicity under at least one condition. However, different TF families showed distinct characteristics. Approximately 20–30% of all genes encoding TFs maintained rhythmicity even after transitioning from a diel light cycle to constant illumination (SI Appendix, Fig. S6). Under LLl constant illumination, during free-running conditions, a relatively large proportion of bHLH-TFs and Myb-TFs retained rhythmic expression, whereas fewer HSF-TFs (16.7%) and bZIP-TFs (4.3%) displayed self-sustained rhythms (Fig. 4). The circadian expression properties of bHLH- and Myb-family TFs are well documented in various organisms, including plants, insects, and mammals (36–39). Regarding cell division, DNA replication, cyclin, cell cycle, and mitosis represented the groups with the highest proportions of genes with self-sustained rhythms under the LL conditions. In contrast, genes encoding cyclin-dependent kinase (CDK) and anaphase-promoting complex subunits, which are involved in signal transduction and proteolytic processes, displayed a higher number of arrhythmic genes, suggesting relatively constitutive expression patterns. These results align with the established mechanism of the cell cycle, where progression is controlled by the interaction between cyclins and CDKs. CDK levels generally remain constant, while their activity is regulated by the presence of cyclin partners. The fluctuating abundance of various cyclins is controlled by the presence of cyclin partners. The fluctuating abundance of various cyclins, governed by their synthesis and proteolysis, is recognized as an intrinsic oscillator that drives the progression of the cell cycle (40).

In contrast to transcriptional regulation and cell division, which showed similar results under both LL_s_ and LL_l_ conditions, photosynthetic processes, including the electron transfer chain (ETC), chlorophyll and carotenoid biosynthesis, and carbon fixation processes, including the carbon concentrating mechanism (CCM) and the Calvin–Benson–Bassham (CBB) cycle, exhibited significant differences between the entrained and nonentrained cultures. These biological processes comprised a high percentage of rhythmic genes among all genes involved. Many genes within these processes demonstrated cyclic expression even after 24 h of constant illumination, suggesting that light harvesting and carbon conversion processes are governed by light-independent circadian rhythms. A microarray study on *Chlamydomonas reinhardtii* identified 269 circadian rhythmic genes, which showed implications in various functions, including photosynthesis, respiration, cellular structure, and diverse metabolic pathways (29). The oscillatory expression of genes involved in photosynthesis and carbon fixation may directly impact biomass production by periodically constraining cell growth. However, after LLl cultivation under constant light conditions, this rhythmic expression was no longer observed. This could be due to the desynchronization of cultures or their adaptation to constant illumination.

The data derived from LL_l_ conditions revealed the presence of a persistent oscillator related to the cell division cycle, which may contribute to the controlled pace of cell growth under constant light conditions. Classical theory suggests that cell growth and cell division are coordinated to maintain cell size homeostasis (41). While environmental factors influence cell growth, it is more likely that the cell cycle is regulated by a size-sensing mechanism rather than a timing-based mechanism (41). The “sizer” model, proposed for various cells from bacteria to animal cells (42–44), has yet to be thoroughly explored in microalgae. Additionally, a homologous gene family containing 10 Myb genes, including Myb3R1 (Phatr3_J15016) and Myb3R2 (Phatr3_J6839), based on orthologous annotations, suggests a potential role in regulating cell proliferation. In *Arabidopsis thaliana*, the Myb3R TFs were found to act as transcriptional repressors to regulate G2/M-specific genes, thereby contributing to the establishment of a post-mitotic quiescent state (45). The intrinsic expression rhythms of these TFs may underlie the circadian rhythms observed in various cell division genes.

### Effect of light quality on circadian rhythm

**Fig. 5.**
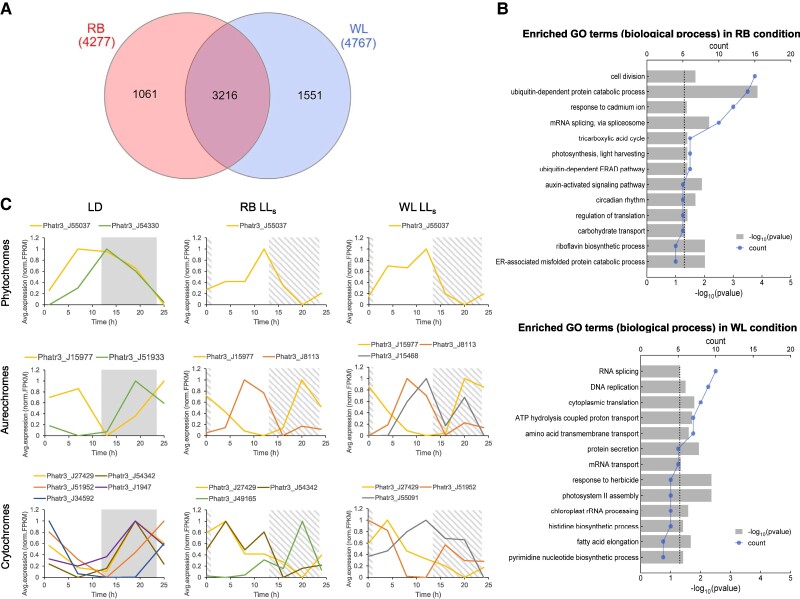
Effects of light quality on circadian rhythms. A) Venn diagram of the statistic of rhythmic genes in red-blue (RB) light and WL conditions. B) GO enrichment analyses on unique genes in RB and WL conditions. C) Rhythmic expression of photoreceptors in different conditions. LD, light/dark cycle; LL_s_, short-term constant light; black squares indicate dark periods in the LD cycle; striped squares indicate subjective dark periods in the LL_s_ condition.

In addition to the LL_s_ group under red-blue (RB) light conditions, another set of cultures was grown under white light (WL), which includes the entire spectrum of visible light. These experiments aimed to investigate the influence of light quality on the expression of rhythmic genes. Similar analyses to those described in the previous sections were performed, identifying 4,767 rhythmic genes in the WL group. A comparison between the RB and WL conditions revealed that 3,216 genes were commonly rhythmic in both groups. Additionally, 1,061 genes were exclusively rhythmic under RB condition, while 1,551 genes were unique to the WL group (Fig. 5A). We anticipated that WL, which covers a broader spectrum and affects a wider range of photoreceptors, would have more extensive impacts by resetting cellular rhythms. However, the relatively large number of rhythmic genes in the WL group was unexpected. The unique genes from each condition were subjected to the GO analysis (Fig. 5B). Compared with the WL group, the RB condition exhibited enrichment of unique rhythmic genes associated with notable terms such as cell division, light harvesting, circadian rhythm, and auxin-activated signaling pathway. Interestingly, the circadian rhythms of these biological processes persisted even without the green spectrum, suggesting that these genes were not reset or repressed in the absence of green light. This indicates that they may be specifically regulated by green light photoreceptors in diatoms.

Photoreceptors that sense the light signals play a crucial role in regulating gene expression. In diatoms, well-documented photoreceptors include red/far-red light photoreceptors called phytochromes (PHY), as well as blue light receptors known as cryptochromes (CRY), and aureochromes (AUREO) (46). However, diatom genomes lack sequences encoding green light photoreceptors, except for bacterial-like (proteo) rhodopsins, which have been characterized as light-driven proton pumps in *Pseudonitzschia granii* and *Fragilariopsis cylindrus* (47–49). We searched for putative photoreceptors in *P. tricornutum* and identified two PHYs, four AUREOs, and eight CRYs. Upon examining their rhythmic characteristics under different conditions, we found that nine photoreceptor genes displayed diel rhythms under the LD condition (Fig. 5C). Previous studies reported diurnal expression of CPF1, CPF2, CPF3, AUREO1c, and Dph in *Thalassiosira pseudonana*, whereas AUREO1a and AUREO2 showed weak control by the LD cycle (48, 50). In general, the transcript abundance of two AUREOs (PtAUREO1b, gene ID: Phatr3_J15977 and PtAUREO1c, gene ID: Phatr3_J51933) and five CRYs increased at night and decreased during the day. This phenomenon was also observed in *Nannochloropsis oceanica*, which exhibited diel expression of AUREO2/3/4 and CPF1 (23). Conversely, the two identified PHYs (Dph1, Phatr3_J54330, and SKP3, Phatr3_J55037) showed opposite expression patterns. Among the photoreceptors, one PHY (Phatr3_J55037), two AUREOs (Phatr3_J15977, Phatr3_J8113), and one CRY (Phatr3_J27429) exhibited cyclic expression under both RB and WL illumination conditions, suggesting light-independent rhythms in their expression. In contrast, the RB condition differed from the WL condition in rhythmic expression of several AUREOs and CRYs, including PtAUREO2 (Phatr3_J15468), CPD1 (Phatr3_J51952), CRYL2 (Phatr3_J54342), CPF4 (Phatr3_J55091), and Phatr3_J49165.

### Effect of constant light on gene expression

**Fig. 6.**
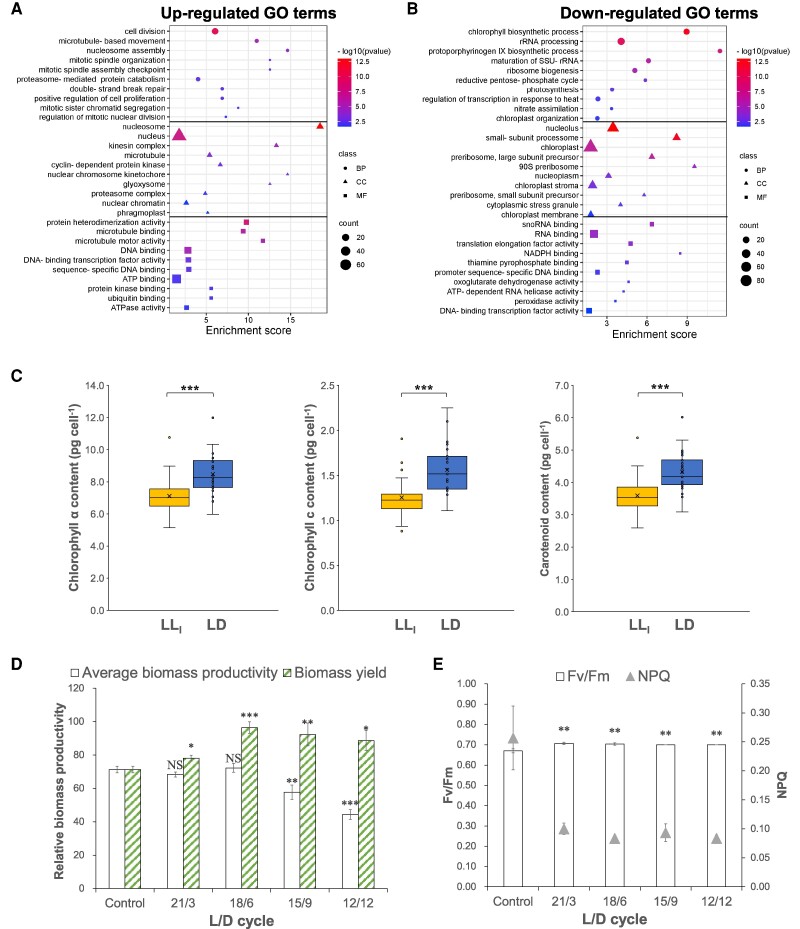
Differential gene expression and contents of pigments in the long-term constant light (LL_l_) group compared with the LD cycle group. A) Enrichment of GO terms based on up-regulated DEGs. B) Enrichment of GO terms based on down-regulated DEGs. An overall differential gene expression profile between the LD and LL_l_ groups was assessed based on data over a 24-h time course with five different time points (*n* = 15). Top enriched GO terms in each category (BP, biological process; CC, cellular component; MF, molecular function) are presented. C) Comparison of estimated pigment contents between cultures under LD and LL_l_ conditions. Samples were collected in 3 h intervals over 24 h time course (*n* = 27). D) Effect of LD cycles on biomass productivity. E) Effect of LD cycles on photosynthetic efficiency. (NS, no significance; *P-value < 0.05; **P-value < 0.01; ***P-value < 0.001).

In this study, we observed that the loss of entrainment under constant light conditions led to arrhythmic expression and significantly reduced oscillations in gene expression compared with the LD condition. This finding suggests that different light regimes can influence cell growth by altering the overall gene expression levels. To explore this hypothesis, differential expression analysis was conducted to compare overall gene expression levels in the two experimental groups across all sampling times. Using the LD group as the control, differentially expressed genes (DEGs) were identified in the LL_l_ group, including 507 up-regulated DEGs and 935 down-regulated DEGs (*P*_adj_ < 0.05). As shown in the GO enrichment results (Fig. 6A and B), top up-regulated biological process GO terms were related to cell division, including kinesin complex, microtubule, and nuclear chromosome kinetochore, which are essential components of mitotic machinery. In contrast, the down-regulated DEGs revealed several key biological processes that are crucial for cell growth and metabolism, including chlorophyll biosynthetic process, ribosome biogenesis, reductive pentose–phosphate cycle (i.e. the CBB cycle), photosynthesis, nitrate assimilation, and carotenoid biosynthetic process (Fig. 6B). The down-regulation of these processes suggests that constant light conditions may impair cell growth by disrupting essential metabolic pathways and photosynthetic activities.

In this study, we identified significant differences in the expression of TFs between the LD and LL_l_ conditions. Specifically, three bHLH-TFs, eight Myb-TFs, five bZIP-TFs, and five HSP-TFs were significantly up-regulated under LL_l­_ compared with the LD condition (SI Appendix, Fig. S7). Conversely, 16 stress-induced HSP-TFs were significantly down-regulated under LL_l_. This pattern suggests higher intracellular stress under the LD condition, likely due to the oscillatory environmental changes. Up-regulated genes were predominantly found in functional groups related to cell division (SI Appendix, Fig. S8), while pathways associated with chlorophyll and carotenoid biosynthesis showed only down-regulated DEGs. Notably, 17 out of 38 chlorophyll biosynthetic genes were down-regulated under the LL_l_ condition, affecting the entire pathway from L-glutamate to chlorophyll *a* (SI Appendix, Fig. S9). Additionally, five genes involved in the synthesis of lycopene, the precursor of carotene from geranyl-geranyl-PP, were significantly down-regulated (SI Appendix, Fig. S10). These results were corroborated by pigment analysis, which revealed significantly higher levels of chlorophylls and total carotenoids per cell under the LD condition compared with LL_l_ (Fig. 6C). While most genes in the ETC and CCM pathways did not show significant changes in expression, eight genes within the CBB cycle were significantly down-regulated (SI Appendix, Fig. S11), indicating a reduction in carbon fixation. The constantly low expression of essential pathways in pigment biosynthesis and CO_2_ assimilation under constant light suggests a decreased photosynthetic efficiency in diatoms. This reduction is likely due to disruption of the regular diel rhythms, which negatively impacts overall gene expression related to photosynthesis and, ultimately hinders diatom growth.

To further demonstrate the importance of the dark period in maintaining photosynthetic efficiency, an experiment was conducted to examine cell growths under different LD cycles. We observed that reducing the photoperiod to 15 and 12 h of illumination only gained 81 ± 6% and 62 ± 4% of the biomass achieved under 24-h photoperiod (control), respectively. However, the average biomass productivities from the 21:3 and 18:6 LD cycles were statistically indistinguishable from the control. When we adjusted the calculations to consider only the light periods, all experimental groups with 3 to 12-h dark periods showed a 10 to 35% higher (*P* < 0.05) biomass yield per unit of light energy compared with the control (Fig. 6D). These results suggest that short dark periods could enhance biomass yield during the light periods. Additionally, the control group exhibited significantly lower maximal quantum yield (Fv/Fm) and higher nonphotochemical quenching (NPQ). The inclusion of a dark period effectively improved these metrics suggesting that periodic darkness enhances overall photosynthetic efficiency in diatoms.

### Differential expression and targeting analysis of microRNA

**Fig. 7.**
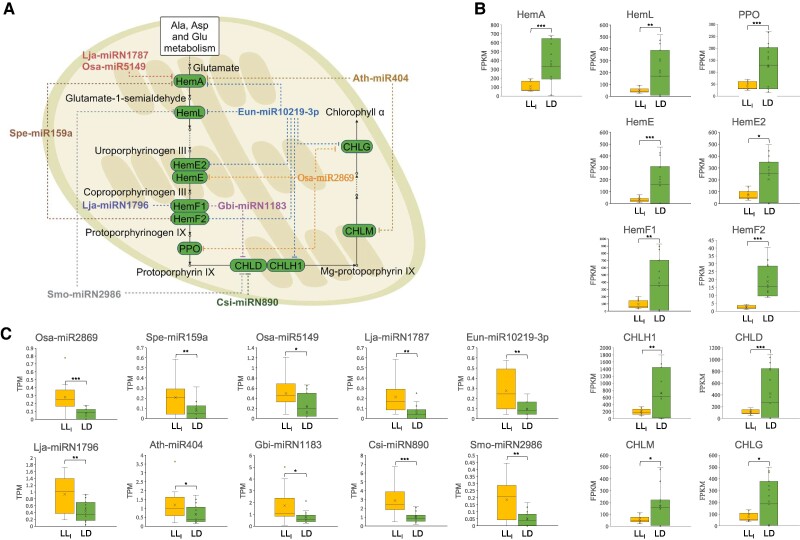
Potential mRNA targets of differentially expressed miRNA under constant light conditions. Differential expression analysis using all data across five sampling times covering 24 h was performed to compare gene expression levels between the long-term constant light (LL_l_) group compared with the LD cycle group. DEGs with significant differences (*P*_adj_ < 0.05) and large fold changes (|log2foldchange| > 1) were screened. A) Pathways in chlorophyll biosynthesis that were possibly targeted by differentially expressed miRNA within 24 h. B) DEG expression levels of coding RNAs targeted by miRNAs. C) DEG expression levels of miRNAs. LL_l_ and LD are presented in yellow and green, respectively. (*P-value < 0.05; **P-value < 0.01; ***P-value < 0.001).

Small RNAs were analyzed to investigate possible microRNA (miRNA)-mediated regulation of gene expression. Rhythmic analysis indicated significant cyclic expression patterns for eight miRNAs in the LL_l_ group and 80 miRNAs in the LD group (SI Appendix, Dataset S5). However, due to large variations among biological replicates, the patterns of rhythmic expression of these miRNAs were not as clear as the genes (mRNAs). This was probably due to the vulnerabilities of miRNA to degradation during the sample processing. Through differential expression analysis on a 24-h course expression of miRNA transcripts in different conditions, 82 differentially expressed miRNAs were identified, including 18 up-regulated mRNAs and 64 down-regulated miRNAs in the LL_l_ group compared with the LD group. Target prediction suggested 751 potential mRNA targets for the up-regulated miRNAs and 1,281 for the down-regulated ones (SI Appendix, Table S3). While miRNAs could match and thus regulate many mRNAs, some mRNAs were targets of multiple miRNAs. Further research was focused on 424 genes (Fig. 4) associated with global transcriptional regulation and cell growth, as previously mentioned. Regarding miRNA-mediated gene silencing through mRNA cleavage (51), the expression levels of mRNAs should theoretically be inversely related to the expression levels of their corresponding miRNAs. However, target identification revealed potential matches of miRNAs with both up-regulated and down-regulated mRNAs (SI Appendix, Table S3). In this study, we specifically explored the coupling of miRNAs and mRNAs with reverse expression levels, whereas other possible targets, such as those regulated during translation or part of more complex regulatory networks involving other small RNAs were not examined here due to a lack of available data. For the coding RNAs of interest, the expression of 47 up-regulated mRNAs and 40 down-regulated mRNAs may be influenced by the differentially expressed miRNAs determined in the present study (SI Appendix, Table S3). For instance, our results revealed 10 up-regulated miRNAs under constant light, which could repress the expression of 11 enzyme-coding genes in the chlorophyll biosynthesis pathway (Fig. 7).

## Materials and methods

### Strain and culture condition

Axenic culture of *Phaeodactylum tricornutum* CCAP 1055/1 was purchased from the Culture Collection of Algae & Protozoa (CCAP), Scotland, UK, and had been incubated under continuous illumination (white fluorescent lamp) for two years. The diatoms were grown in filter-sterilized ESAW (Enriched Seawater, Artificial Water) (52) media containing 100 μg mL^−1^ ampicillin, kanamycin, and streptomycin. The culture pH was maintained at 8.00 ± 0.10 by supplying 40 mM Tris–HCl buffer in the media. For experiments, triplicated cultures in 500 mL flasks (200 mL working volume) were cultivated under combined red (630 ± 20 nm) and blue (450 ± 20 nm) LED panels (50:50), which were frequently adopted for efficient algal cultivation or white LED at a light intensity of 40.0 ± 2.0 μmol m^−2^s^−1^ (as a control for comparison) and the temperature was maintained at 20 ± 2 °C. Seed culture was prepared in batch culture and collected during the exponential growth phase.

### Experiment A

Before the experiment, two groups of inoculums were acclimated to constant light (LL) and a diel cycle of 12 h light:12 h darkness (LD) photoperiod (lights on at 7 AM; lights off at 7 PM) for two weeks. Experiments were conducted in semi-continuous cultivation. Cell growths were monitored by measuring optical density at 750 nm (OD_750_) every 3 h using a DR3900 spectrophotometer (Hach, US) (SI Appendix, Dataset S1). When cell density reached OD_750_ = 0.25, cultivation was swapped to semi-continuous mode to maintain a constant cell density and culture volume through regularly diluting and removing part of the media. Meanwhile, chlorophyll fluorescence was measured to assess photosynthetic performance. Samples for RNA sequencing were collected 1 h after the light was turned on in the morning. Samples (40 mL) were collected every 6 h by centrifugation at 4,000*×g* for 10 min, which covered 24 h with five time points. Cell pellets were washed once using sterile Milli-Q water and stored in 1 mL of sample protector reagent (Takara, Japan) at −20 °C until RNA sequencing.

### Experiment B

Two groups of triplicated culture were cultivated under white and 50:50 red-blue LED (40 μmol m^2^ s^−1^) conditions, respectively, and entrained through 12:12 LD cycles until the OD_750_ reached 0.25. Then, illumination was swapped to constant condition, and the cultures were grown using semi-continuous cultivation by diluting with 10 mL of fresh ESAW media at 2-h intervals. At 4-h intervals, 20 mL of cell culture was removed. After the first 24 h under constant illumination, 20 mL of culture was collected every 4 h. Pelleted cell samples were frozen using liquid nitrogen and stored at −20 °C until RNA sequencing.

### Calculation of growth rate and productivity

Ash-free dry weight (AFDW) of *P. tricornutum* was measured according to Zhu and Lee (53). The relationship between AFDW and OD_750_ was established as following, AFDW (mg L^−1^) = 415.1OD_750_ (*R*^2^ = 0.9915). The specific growth rate (*μ*, d^−1^) was calculated using the following equation: $\mu =(\mathrm{In}X_{t}-\;\mathrm{In}X_{0})/(t-t_{0})$ where *X_t_* and *X*_0_ are the OD at time *t* and the beginning of the time interval (*t*_0_), respectively (SI Appendix, Dataset S1). As AFDW was estimated based on OD data, the average biomass productivity (P_b_, mg L^−1^d^−1^) in a period (*t*) was calculated as accumulated biomass divided by time, P_b_ = ΔAFDW/*t*.

### Chlorophyll fluorescence measurements

To assess photosynthetic efficiency, a 3 mL sample was incubated in the dark for 15 min. Chlorophyll fluorescence was measured (SI Appendix, Dataset S1) using an AquaPen-C (Photon Systems Instruments, Czech Republic) according to a built-in program with (NPQ) protocol 3 that generated maximum quantum yield (Fv/Fm) and effective quantum yield (Φ_PSII_).

### Pigment content assay

During semi-continuous cultivation, aliquots of 2 mL were collected in 3 h intervals. The cell pellet was produced by centrifugation at 4,000*×g* for 10 min at 4 °C. Pigments were extracted by adding 1 mL of 100% ethanol to the cell pellet, followed by sonication for 2 min. Cell debris was pelleted by centrifugation at 12,000*×g* for 10 min (4 °C) and the supernatant was transferred to a cuvette for spectrophotometric assay at 630, 664, and 470 nm using a DR3900 spectrophotometer (Hach, US). Concentrations of pigments including chlorophyll a, chlorophyll c_1_+c_2_, and total carotenoids were estimated using equations (54, 55) and then normalized based on the cell concentration of each sample measured using a hematocytometer to obtain the value of pigment contents per cell.

### Transcriptomics

Rhythmicity and differential gene expression were revealed based on transcriptome profiling. RNA sequencing was conducted by OE Biotech Co. Ltd. (Shanghai, China). More details of RNAseq and bioinformatic analyses are described in the SI Appendix.

### Statistical analysis

Statistical significance of physiological data was assessed using a two-tailed Student's t test on Microsoft Excel. All data include more than three independent biological replicates and are reported as mean ± SD. Default statistical analyses in the JTK and DEseq R packages were used for rhythm detection and differential expression analyses.

## Supplementary Material

pgae497_Supplementary_Data

## Data Availability

All data needed to evaluate the conclusions are present in the paper and/or SI Appendix. All the original RNA-seq data were deposited to the National Centre for Biotechnology Information (NCBI) database with an accession number of PRJNA922714 and PRJNA1100328.

## References

[1] Filonova A, Haemsch P, Gebauer C, Weisheit W, Wagner V. 2013. Protein disulfide isomerase 2 of *Chlamydomonas reinhardtii* is involved in circadian rhythm regulation. Mol Plant. 6:1503–1517. 23475997 10.1093/mp/sst048

[2] Greenham K, McClung CR. 2015. Integrating circadian dynamics with physiological processes in plants. Nat Rev Genet. 16:598–610. 26370901 10.1038/nrg3976

[3] Farré EM . 2020. The brown clock: circadian rhythms in stramenopiles. Physiol Plant. 169:430–441. 32274814 10.1111/ppl.13104

[4] Tanaka K, et al 2019. The endogenous redox rhythm is controlled by a central circadian oscillator in cyanobacterium *Synechococcus elongatus* PCC7942. Photosynth Res. 142:203–210. 31485868 10.1007/s11120-019-00667-0

[5] Dodd AN, Kusakina J, Hall A, Gould PD, Hanaoka M. 2014. The circadian regulation of photosynthesis. Photosynth Res. 119:181–190. 23529849 10.1007/s11120-013-9811-8

[6] Wright KP Jr, et al 2013. Entrainment of the human circadian clock to the natural light-dark cycle. Curr Biol. 23:1554–1558. 23910656 10.1016/j.cub.2013.06.039 PMC4020279

[7] Chauton MS, Winge P, Brembu T, Vadstein O, Bones AM. 2013. Gene regulation of carbon fixation, storage, and utilization in the diatom *Phaeodactylum tricornutum* acclimated to light/dark cycles. Plant Physiol. 161:1034–1048. 23209127 10.1104/pp.112.206177 PMC3561001

[8] Arrigo KR, van Dijken GL. 2011. Secular trends in Arctic Ocean net primary production. J Geophys Res Oceans. 116:C09011.

[9] Lewis K, Van Dijken G, Arrigo KR. 2020. Changes in phytoplankton concentration now drive increased Arctic Ocean primary production. Science 369:198–202. 32647002 10.1126/science.aay8380

[10] Terhaar J, Lauerwald R, Regnier P, Gruber N, Bopp L. 2021. Around one third of current Arctic Ocean primary production sustained by rivers and coastal erosion. Nat Commun. 12:169. 10.1038/s41467-020-20470-z 33420093 PMC7794587

[11] Friend AD, Geider RJ, Behrenfeld MJ, Still CJ. 2009. Photosynthesis in global-scale models. In: Laisk A, Nedbal L, Govindjee, editors. Photosynthesis In Silico: understanding complexity from molecules to ecosystems. Dordrecht: Springer. p. 465–497.

[12] Beer C, et al 2010. Terrestrial gross carbon dioxide uptake: global distribution and covariation with climate. Science 329:834–838. 20603496 10.1126/science.1184984

[13] Welp LR, et al 2011. Interannual variability in the oxygen isotopes of atmospheric CO2 driven by El Niño. Nature 477:579–582. 21956330 10.1038/nature10421

[14] Sirisuk P, Ra C-H, Jeong G-T, Kim S-K. 2018. Effects of wavelength mixing ratio and photoperiod on microalgal biomass and lipid production in a two-phase culture system using LED illumination. Bioresour Technol. 253:175–181. 29348061 10.1016/j.biortech.2018.01.020

[15] Wahidin S, Idris A, Shaleh SRM. 2013. The influence of light intensity and photoperiod on the growth and lipid content of microalgae *Nannochloropsis* sp. Bioresour Technol. 129:7–11. 23232218 10.1016/j.biortech.2012.11.032

[16] de Winter L, Klok AJ, Franco MC, Barbosa MJ, Wijffels RH. 2013. The synchronized cell cycle of *Neochloris oleoabundans* and its influence on biomass composition under constant light conditions. Algal Res. 2:313–320.

[17] Poliner E, et al 2015. Transcriptional coordination of physiological responses in *Nannochloropsis oceanica* CCMP1779 under light/dark cycles. Plant J. 83:1097–1113. 26216534 10.1111/tpj.12944

[18] Pabi S, van Dijken GL, Arrigo KR. 2008. Primary production in the Arctic Ocean, 1998–2006. J Geophys Res Oceans. 113:C08005.

[19] Arrigo KR, van Dijken GL, Bushinsky S. 2008. Primary production in the Southern Ocean, 1997–2006. J Geophys Res Oceans. 113:C08004.

[20] Arrigo KR, Worthen D, Schnell A, Lizotte MP. 1998. Primary production in Southern Ocean waters. J Geophys Res Oceans. 103:15587–15600.

[21] Ragni M . 2005. Circadian patterns in key physiological processes of the marine diatom Phaeodactylum tricornutum [PhD thesis]. The Open University.

[22] de Winter L, Cabanelas ITD, Martens DE, Wijffels RH, Barbosa MJ. 2017. The influence of day/night cycles on biomass yield and composition of *Neochloris oleoabundans*. Biotechnol Biofuels. 10:104. 28439297 10.1186/s13068-017-0762-8 PMC5401387

[23] Poliner E, et al 2015. Transcriptional coordination of physiological responses in *Nannochloropsis oceanica* CCMP 1779 under light/dark cycles. Plant J. 83:1097–1113. 26216534 10.1111/tpj.12944

[24] Bilcke G, et al 2021. Diurnal transcript profiling of the diatom *Seminavis robusta* reveals adaptations to a benthic lifestyle. Plant J. 107:315–336. 33901335 10.1111/tpj.15291

[25] Ferrari C, et al 2019. Kingdom-wide comparison reveals the evolution of diurnal gene expression in Archaeplastida. Nat Commun. 10:737. 30760717 10.1038/s41467-019-08703-2 PMC6374488

[26] Smith SR, et al 2016. Transcriptional orchestration of the global cellular response of a model pennate diatom to diel light cycling under iron limitation. PLoS Genet. 12:e1006490. 27973599 10.1371/journal.pgen.1006490 PMC5156380

[27] Zones JM, Blaby IK, Merchant SS, Umen JG. 2015. High-resolution profiling of a synchronized diurnal transcriptome from *Chlamydomonas reinhardtii* reveals continuous cell and metabolic differentiation. Plant Cell. 27:2743–2769. 26432862 10.1105/tpc.15.00498 PMC4682324

[28] Annunziata R, et al 2019. bHLH-PAS protein RITMO1 regulates diel biological rhythms in the marine diatom *Phaeodactylum tricornutum*. Proc Natl Acad Sci U S A. 116:13137–13142. 31171659 10.1073/pnas.1819660116 PMC6600994

[29] Kucho K-i, Okamoto K, Tabata S, Fukuzawa H, Ishiura M. 2005. Identification of novel clock-controlled genes by cDNA macroarray analysis in *Chlamydomonas reinhardtii*. Plant Mol Biol. 57:889–906. 15952072 10.1007/s11103-005-3248-1

[30] Moulager M, et al 2007. Light-dependent regulation of cell division in ostreococcus: evidence for a major transcriptional input. Plant Physiol. 144:1360–1369. 17535824 10.1104/pp.107.096149 PMC1914124

[31] Xu Y, Ibrahim IM, Harvey PJ. 2016. The influence of photoperiod and light intensity on the growth and photosynthesis of *Dunaliella salina* (chlorophyta) CCAP 19/30. Plant Physiol Biochem. 106:305–315. 27231875 10.1016/j.plaphy.2016.05.021 PMC5250801

[32] Ragni M, d'Alcalà MR. 2007. Circadian variability in the photobiology of *Phaeodactylum tricornutum*: pigment content. J Plankton Res. 29:141–156.

[33] Thaben PF, Westermark PO. 2014. Detecting rhythms in time series with RAIN. J Biol Rhythms. 29:391–400. 25326247 10.1177/0748730414553029 PMC4266694

[34] Monnier A, et al 2010. Orchestrated transcription of biological processes in the marine picoeukaryote *Ostreococcus* exposed to light/dark cycles. BMC Genomics. 11:192. 20307298 10.1186/1471-2164-11-192 PMC2850359

[35] Bulankova P, Bilcke G, Vyverman W, Veylder LD. 2022. Cellular hallmarks and regulation of the diatom cell cycle. In: Falciatore A, Mock T, editors. The molecular life of diatoms. Cham: Springer International Publishing. p. 229–263.

[36] Lim C, et al 2007. Clockwork orange encodes a transcriptional repressor important for circadian-clock amplitude in *Drosophila*. Curr Biol. 17:1082–1089. 17555964 10.1016/j.cub.2007.05.039 PMC1963421

[37] Nguyen NH, Lee H. 2016. MYB-related transcription factors function as regulators of the circadian clock and anthocyanin biosynthesis in Arabidopsis. Plant Signal Behav. 11:e1139278. 26905954 10.1080/15592324.2016.1139278 PMC4883932

[38] Saini R, Jaskolski M, Davis SJ. 2019. Circadian oscillator proteins across the kingdoms of life: structural aspects. BMC Biol. 17:13. 10.1186/s12915-018-0623-3 30777051 PMC6378743

[39] Yamashino T, et al 2003. A link between circadian-controlled bHLH factors and the APRR1/TOC1 quintet in *Arabidopsis thaliana*. Plant Cell Physiol. 44:619–629. 12826627 10.1093/pcp/pcg078

[40] Poon RY . 2016. Cell cycle control: a system of interlinking oscillators. Methods Mol Biol. 1342:3–19. 10.1007/978-1-4939-2957-3_1 26254915

[41] Björklund M . 2019. Cell size homeostasis: metabolic control of growth and cell division. Biochim Biophys Acta Mol Cell Res. 1866:409–417. 30315834 10.1016/j.bbamcr.2018.10.002

[42] Jones AR, et al 2017. Cell-size dependent progression of the cell cycle creates homeostasis and flexibility of plant cell size. Nat Commun. 8:15060. 28447614 10.1038/ncomms15060 PMC5414177

[43] Ginzberg MB, et al 2018. Cell size sensing in animal cells coordinates anabolic growth rates and cell cycle progression to maintain cell size uniformity. Elife. 7:e26957. 29889021 10.7554/eLife.26957 PMC6031432

[44] Robert L, et al 2014. Division in *Escherichia coli* is triggered by a size-sensing rather than a timing mechanism. BMC Biol. 12:17. 24580833 10.1186/1741-7007-12-17 PMC4016582

[45] Kobayashi K, et al 2015. Transcriptional repression by MYB 3R proteins regulates plant organ growth. EMBO J. 34:1992–2007. 26069325 10.15252/embj.201490899 PMC4551348

[46] Jaubert M, Bouly J-P, d’Alcalà MR, Falciatore A. 2017. Light sensing and responses in marine microalgae. Curr Opin Plant Biol. 37:70–77. 28456112 10.1016/j.pbi.2017.03.005

[47] Marchetti A, Catlett D, Hopkinson BM, Ellis K, Cassar N. 2015. Marine diatom proteorhodopsins and their potential role in coping with low iron availability. ISME J. 9:2745–2748. 26023874 10.1038/ismej.2015.74 PMC4817633

[48] Ashworth J, et al 2013. Genome-wide diel growth state transitions in the diatom *Thalassiosira pseudonana*. Proc Natl Acad Sci U S A. 110:7518–7523. 23596211 10.1073/pnas.1300962110 PMC3645528

[49] S. Yoshizawa et al, 20 January 2022. Proton-pumping rhodopsins in marine diatoms. BioRxiv 476826. 10.1101/2022.01.18.476826, preprint: not peer reviewed.

[50] Goldman JA, et al 2019. Fe limitation decreases transcriptional regulation over the diel cycle in the model diatom *Thalassiosira pseudonana*. PLoS One. 14:e0222325. 31509589 10.1371/journal.pone.0222325 PMC6738920

[51] Waititu JK, Zhang C, Liu J, Wang H. 2020. Plant Non-Coding RNAs: origin, biogenesis, mode of action and their roles in abiotic stress. Int J Mol Sci. 21:8401. 33182372 10.3390/ijms21218401 PMC7664903

[52] Berges JA, Franklin DJ, Harrison PJ. 2001. Evolution of an artificial seawater medium: improvements in enriched seawater, artificial water over the last two decades. J Phycol. 37:1138–1145.

[53] Zhu C, Lee Y. 1997. Determination of biomass dry weight of marine microalgae. J Appl Phycol. 9:189–194.

[54] Ritchie RJ . 2006. Consistent sets of spectrophotometric chlorophyll equations for acetone, methanol and ethanol solvents. Photosynth Res. 89:27–41. 16763878 10.1007/s11120-006-9065-9

[55] Ryckebosch E, Muylaert K, Eeckhout M, Ruyssen T, Foubert I. 2011. Influence of drying and storage on lipid and carotenoid stability of the microalga *Phaeodactylum tricornutum*. J Agric Food Chem. 59:11063–11069. 21866882 10.1021/jf2025456

