# Supplementary material for: An undiscovered circadian clock to regulate phytoplankton photosynthesis
Source: PNAS Nexus. 2024 Nov 6;3(11):pgae497. doi: 10.1093/pnasnexus/pgae497 (PMC11563040; doi:10.1093/pnasnexus/pgae497)
Supplement: pgae497_Supplementary_Data [file pgae497_supplementary_data.zip › PNASNEXUS-PNASNEXUS-2024-00646RR-s09.docx]

**
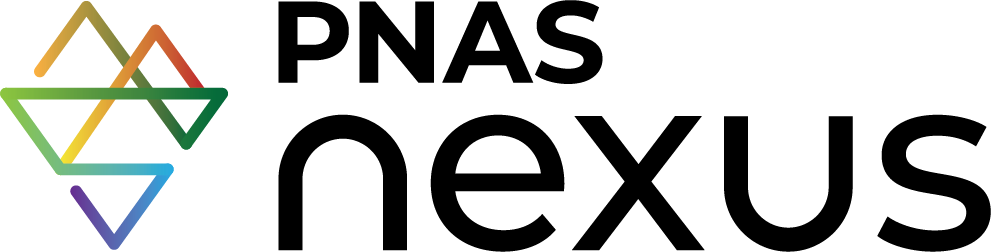
**

An undiscovered circadian clock to regulate phytoplankton photosynthesis

Yixi Su^1,2,3^, Jingyan Hu^1^, Mengsheng Xia^1^, Jiwei Chen^1^, Weizhao Meng^1^, Cheng Qian^1^, Yuexuan Shu^1^, Chao Wang^1^, Xianwei Wang^4^, Kourosh Salehi-Ashtiani^5^, Sigurður Brynjólfsson^3^, Jianping Lin^6^, Yongquan Li^7^, Haisheng Zhang^1,8^, Lizhong Wang^1,9^, Weiqi Fu^1,2,3,10,*^

^1^Ocean College, Zhejiang University, Zhoushan, 316021, Zhejiang, China;

^2^Ocean Research Center of Zhoushan, Zhejiang University, Zhoushan 316021, China;

^3^Center for Systems Biology and Faculty of Industrial Engineering, School of Engineering and Natural Sciences, University of Iceland, Reykjavík 101, Iceland;

^4^School of Oceanography, Shanghai Jiao Tong University, Shanghai 200030, China;

^5^Laboratory of Algal, Systems, and Synthetic Biology, Division of Science and Math & Center for Genomics and Systems Biology, New York University Abu Dhabi, P.O. Box 129188, Abu Dhabi, UAE.

^6^Key Laboratory of Biomass Chemical Engineering of Ministry of Education, College of Chemical and Biological Engineering, Zhejiang University, Hangzhou, 310058, Zhejiang, China;

^7^Institute of Pharmaceutical Biotechnology & Research Center for Clinical Pharmacy, The First Affiliated Hospital, School of Medicine, Zhejiang University, Hangzhou, 310058, Zhejiang, China;

^8^Key Laboratory of Offshore Geotechnics and Material of Zhejiang Province, College of Civil Engineering and Architecture, Zhejiang University, Hangzhou, 310058, Zhejiang, China;

^9^Ocean Academy, Zhejiang University, Zhoushan, 316021, Zhejiang, China;

^10^Donghai Laboratory, Zhoushan, 316021, Zhejiang, China;

*Corresponding author: Weiqi Fu

Email: [weiqifu@zju.edu.cn](mailto:weiqifu@zju.edu.cn) or [weiqi@hi.is](mailto:weiqi@hi.is)

**This PDF file includes:**

Additional Materials and Methods

Supplementary text

Figures S1 to S11

Tables S1 to S3

SI References

**Other supplementary materials for this manuscript include the following:**

Datasets S1 to S8

**Supplementary Information Text**

Materials and Methods

**RNA extraction and sequencing.** Transcriptome sequencing and analysis were conducted by OE Biotech Co. Ltd. (Shanghai, China). Total RNA was extracted using the mirVana miRNA Isolation Kit (Ambion) following the manufacturer’s protocol. RNA integrity was evaluated using the Agilent 2100 Bioanalyzer (Agilent Technologies, Santa Clara, CA, USA) and RT-qPCR. The samples with RNA Integrity Number (RIN) ≥ 7 were subjected to the subsequent analysis. According to the manufacturer's instructions, an amount of 1 ug RNA per sample was used for library construction using TruSeq Stranded Total RNA with Ribo-Zero Gold. Then the constructed libraries were sequenced on the Illumina sequencing platform (HiSeq x-10) and 150 paired-end reads were generated.

**Bioinformatic analyses**. Raw reads were filtered using Trimmomatic(1) to remove adapter sequences, poly-N containing, and low-quality reads. As a result, Experiment A generated 67.8 – 99.9 million reads with a Q30 base > 91.9%. Experiment B generated 37.3 – 49.9 million reads with a Q30 base > 92.6%. The clean reads were mapped to the reference genome using Hisat2(2) and assembled using StringTie(3). While more than 98% of total reads were mapped to the genome for all samples, uniquely mapping rates on average were 65.6% and 66.3% for the LD and LL_l_ groups, respectively, in Experiment A. From Experiment B, > 95% of reads were mapped to the genome, including an average of 93.8% and 93.9% uniquely mapped reads under the red-blue (RB) and white light (WL) conditions, respectively (*SI Appendix,* Dataset S2). The htseq-count (4) was used to count the read abundance of each transcript (protein-coding gene), which was normalized into fragments per kilobase of exon per million mapped fragments (FPKM) using cufflinks(5).

For small RNA, the raw sequence data were processed by removing adaptor sequences, low-quality reads and reads shorter than 15nt and longer than 41nt to generate the data of clean reads. The clean reads were mapped to the genome of *P. tricornutum* using Bowtie (a software package for sequence alignment)(6) (*SI Appendix,* Dataset S3). Non-coding RNAs, including rRNAs, tRNAs, small nuclear RNAs (snRNAs), and small nucleolar RNAs (snoRNAs) were identified using the BLAST(7) search against Rfam v.10.1 (<http://www.sanger.ac.uk/software/Rfam>)(8) and GenBank databases (<http://www.ncbi.nlm.nih.gov/genbank/>) using an e-value of 0.01 as cutoff (*SI Appendix,* Dataset S3). Degraded fragments of mRNAs were identified by aligning reads to exons and introns of annotated mRNAs in the genome of *P. tricornutum*. Additionally, repeat sequences were identified by comparing the clean reads to the Repbase (<https://www.girinst.org/repbase/>). These fractions of RNAs were removed from the clean reads. Then, the bowtie was used to identify microRNAs (miRNAs) by comparing the clean reads with known plant miRNA sequences deposited in the miRBase v.22 database (<http://www.mirbase.org/>)(9) and PmiREN (Plant miRNA Encyclopedia, <https://www.pmiren.com/>) with one mismatch permitted. While between 423 and 1013 miRNAs were identified in LL_l_ samples, the number of identified miRNAs in LD samples varied from 580 to 2251 (*SI Appendix,* Dataset S3). The remaining unannotated small RNAs were analyzed using mirdeep2 (10) to predict novel miRNAs. Based on the hairpin structure of a pre-miRNA and the miRBase database, the corresponding miRNA star sequence was also identified. In total, 38 novel miRNAs within 27 novel miRNA families were revealed across all samples (*SI Appendix,* Dataset S3). The expression levels of miRNAs were normalized by transcript per million (TPM).

Rhythmic genes were detected using the JTK_cycle (MetaCycle R package) and RAIN algorithm based on time course data of triplicated values of expression level (*SI Appendix,* Dataset S4). The target period was set to be 24 h and an adjusted p-value < 0.05 was used to define significant rhythmic transcripts. The detected cycling genes with maximum FPKM > 1, fold change between maximum and minimum FPKM values > 1.3, and amplitude > 1 were further screened to omit lowly expressed and weak oscillating genes (*SI Appendix,* Dataset S5). To assess overall expression in LL_l_ and LD groups, read counts (*SI Appendix,* Dataset S4) derived from five points throughout 24 h were subjected to differential gene screening using the DESeq2 R package (11). With adjusted p-value < 0.05, fold change ratios (LL_l_/LD) ≥ 2 or fold change ratios (LL_l_/LD) ≤ 0.5 were set as the thresholds for significantly differential expression (*SI Appendix,* Dataset S6).

The differentially expressed mRNA and miRNA were analyzed using miRanda (12) with default parameters to predict the potential targeting relationship between mRNA and miRNA. Likely target sites were screened using the parameters as follows: match score S ≥ 90 and target duplex free energy ΔG ≤ -20 kcal mol^-1^ (13).

Gene Ontology (GO) enrichment analysis was performed to assess significant annotations within different clusters of rhythmic genes as well as differentially expressed genes between different light regimes. Annotations of protein-coding genes in *P. tricornutum* were obtained by mapping transcripts to the Swiss-Prot database. The significance of GO term enrichment was examined by a hypergeometric test with a p-value < 0.05.

Concerning cell growth, further research was carried out for specific biological processes of interest, including transcriptional regulation, cell division, photosynthesis, and carbon fixation. Genes for specific functions were listed in *SI Appendix,* Dataset S7. In silico examination indicated that the numbers of putative transcriptional factors with bHLH, Myb, bZIP, and HSF domain in *P. tricornutum* were 9, 39, 23, and 78, respectively. For cell division, we explored 28 cyclins and 17 cyclin-dependent kinases (CDKs) present in *P. tricornutum*. Moreover, genes involved in DNA replication were explored including genes encoding 10 DNA polymerases, 7 DNA mismatch repair proteins, and 10 MCM proteins that serve as DNA replication licensing factors for ensuring the completion of genomic DNA replication(14). Regarding mitosis, 8 SMC-encoding genes that function in mitotic anaphase for chromosome condensation were detected in our transcriptome data. Also, we investigated the expression of 13 KIF and 3 kinetochore proteins for chromosome segregation (15). In addition to 11 genes encoding APC subunits, we investigated other 20 various genes(15) that are presumably involved in cell cycle control. In addition, genes involved in photosynthesis and carbon fixation processes were retrieved from the genome-wide metabolic model of *P. tricornutum*(16). The gene lists for these processes were completed by searching on KEGG and PLAZA databases.


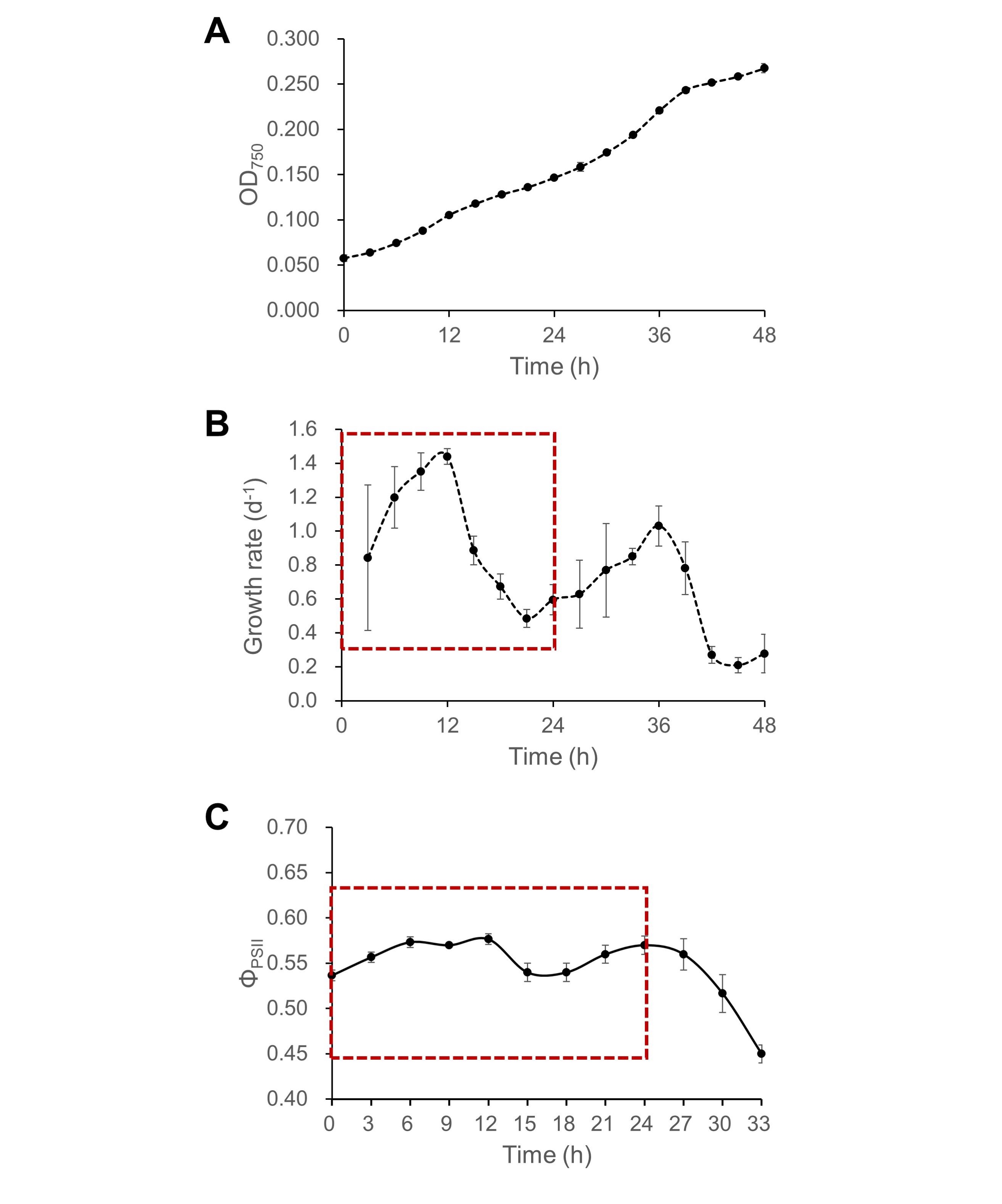


**Fig. S1.** Oscillatory physiology of *P. tricornutum* (non-synchronized culture) during batch cultivation under constant light. *(A)* Growth curve; *(B)* Growth rates at each interval; *(C)* Effective quantum yield (Φ_PSII_).


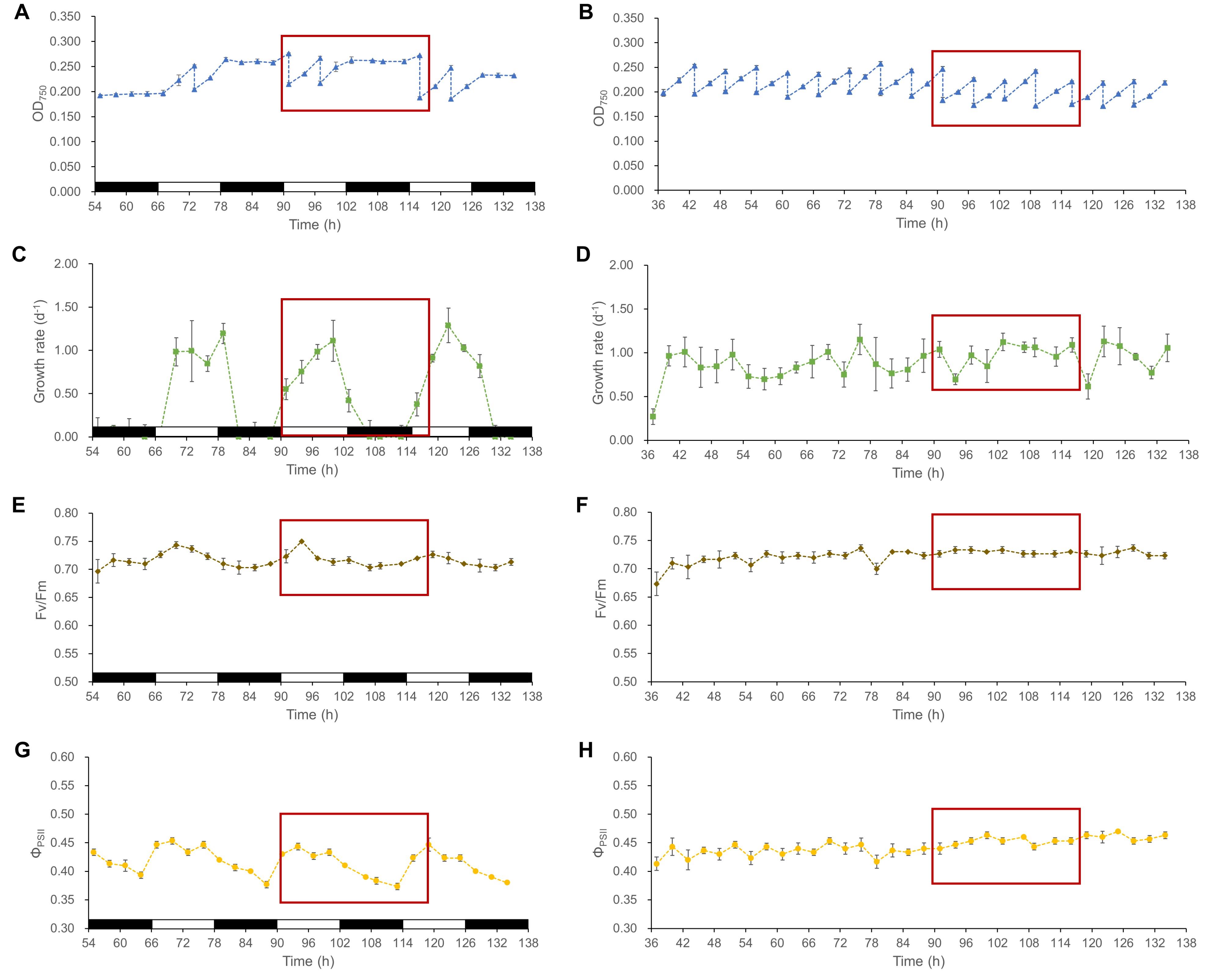


Fig. S2. Semi-continuous cultivation of *P. tricornutum* under the light/dark cycle and constant light condition. *(A)* and *(B)* Cell density (OD_750_); *(C)* and *(D)* Growth rate; *(E)* and *(F)* maximum quantum yield (F_v_/F_m_); *(G)* and *(H)* Effective quantum yield (Φ_PSII_). Samples were measured every three hours with triplicated cultures. Panels (A), (C), (E), and (G) represent the data resulted from light/dark cycle. Panels (B), (D), (F), and (H) represent the data resulted from constant light. Black blocks indicate dark periods. The red rectangles indicate the sampling time for molecular analysis.


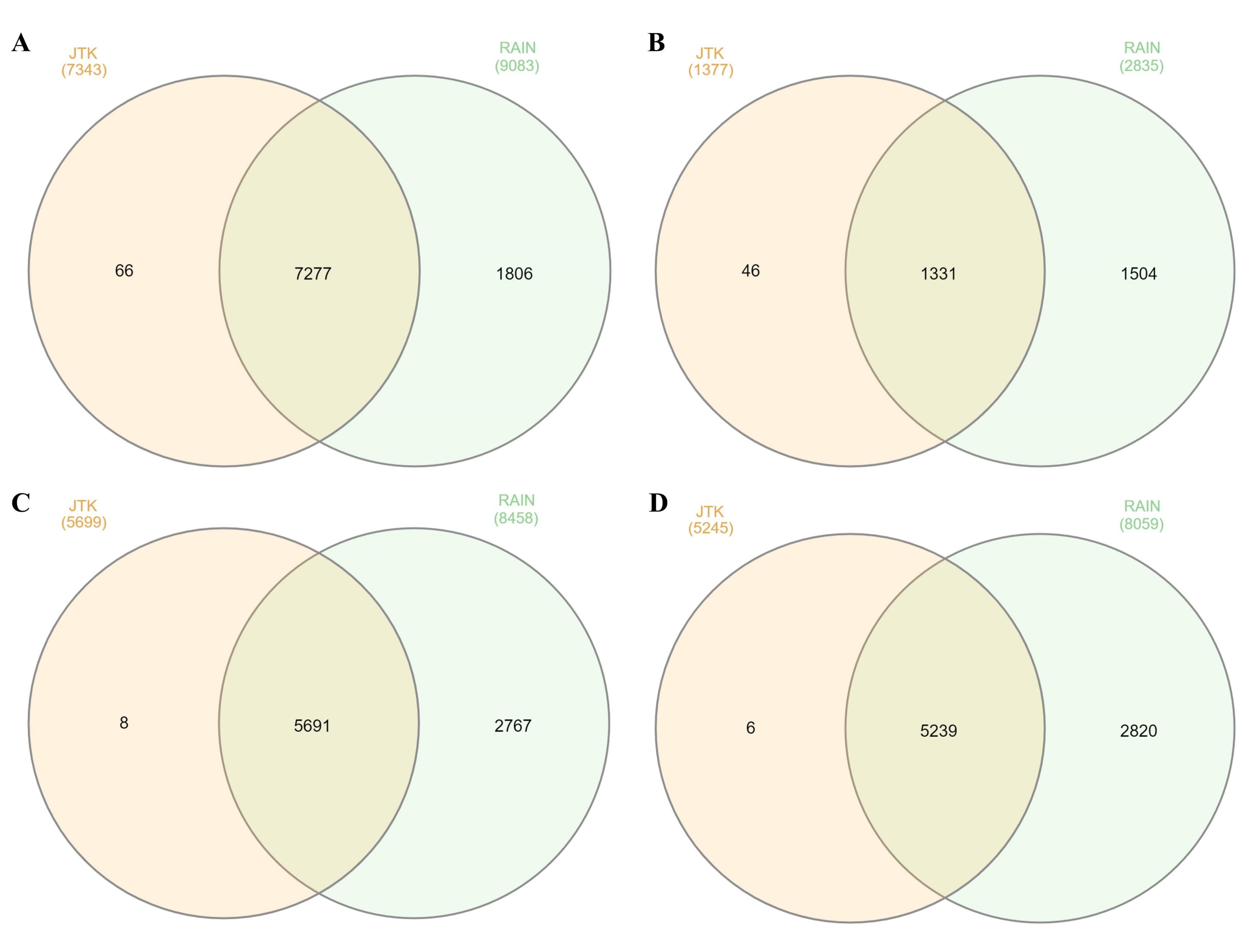


**Fig. S3.** Comparison of results from the JTK and RAIN methods for rhythmic gene detection. *(A)* Light/dark cycle (LD); *(B)* Long-term constant light (LL_l_); *(C)* Short-term constant illumination (LL_s_) under white light (WL); *(D)* Short-term constant illumination (LL_s_) under red-blue light (RB).


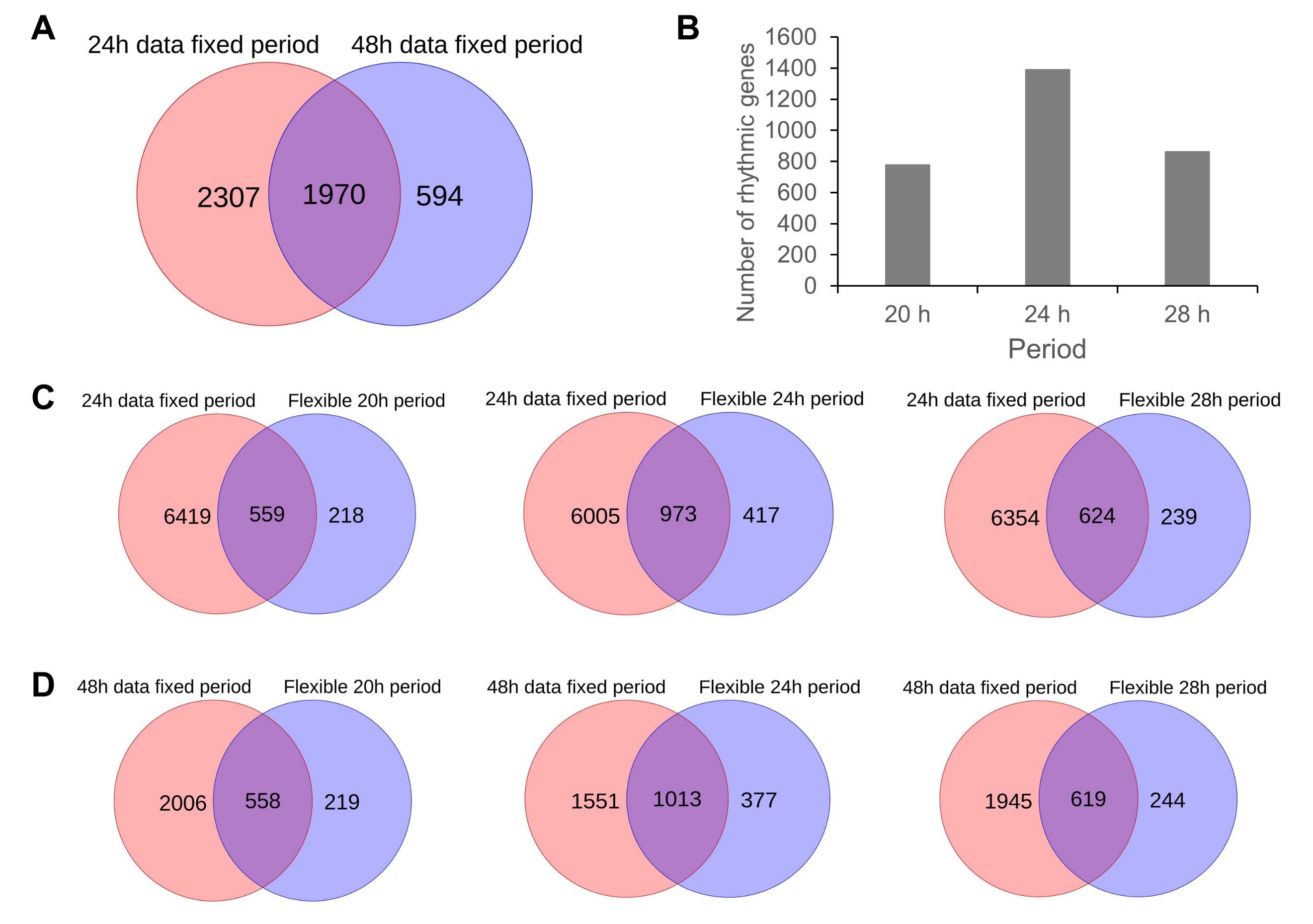


**Fig. S4.** Comparative analysis of rhythm detection across different datasets and period settings. *(A)* Identification of rhythmic genes under constant light (LL) following light-dark (LD) entrainment. The analysis was conducted using both a 24-hour dataset and an extended 48-hour dataset; *(B)* The number of rhythmic genes identified with flexible periods (24±4 hour) using the 48-hour dataset; *(C)* Comparison of rhythmic genes identified with flexible periods to those detected using a fixed 24-hour period in the 24-hour dataset; *(D)* Comparison of rhythmic genes identified with flexible periods to those detected using a fixed 24-hour period in the 48-hour dataset.


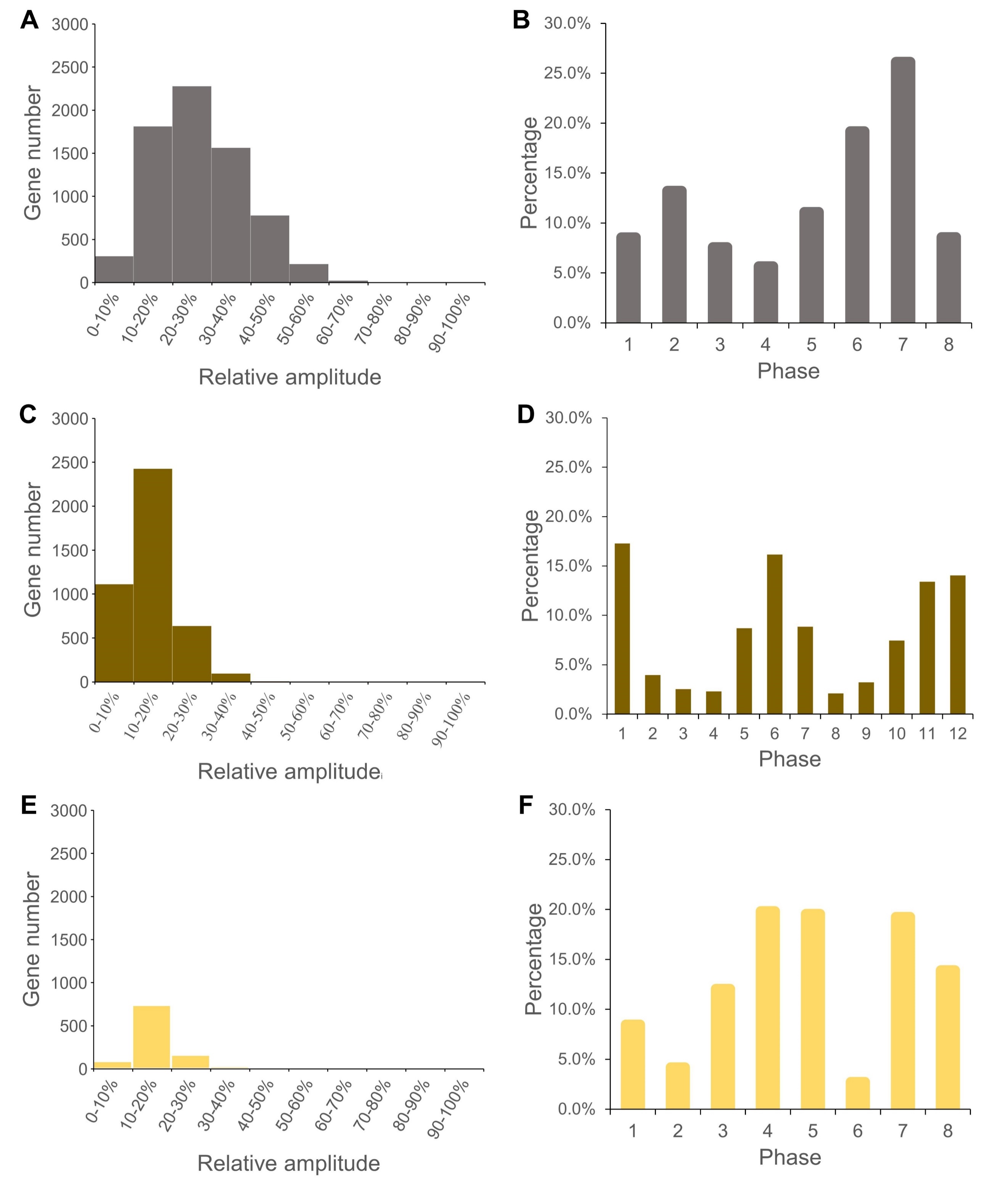


**Fig. S5.** Distributions of amplitudes relative to maximal expression (FPKM) of identified rhythmic genes and phase distribution of rhythmic genes in LL and LD groups. *(A)* and *(B)* LD group. *(C)* and *(D)* LL_s_ group. *(E)* and *(F)* LL_l_ group.


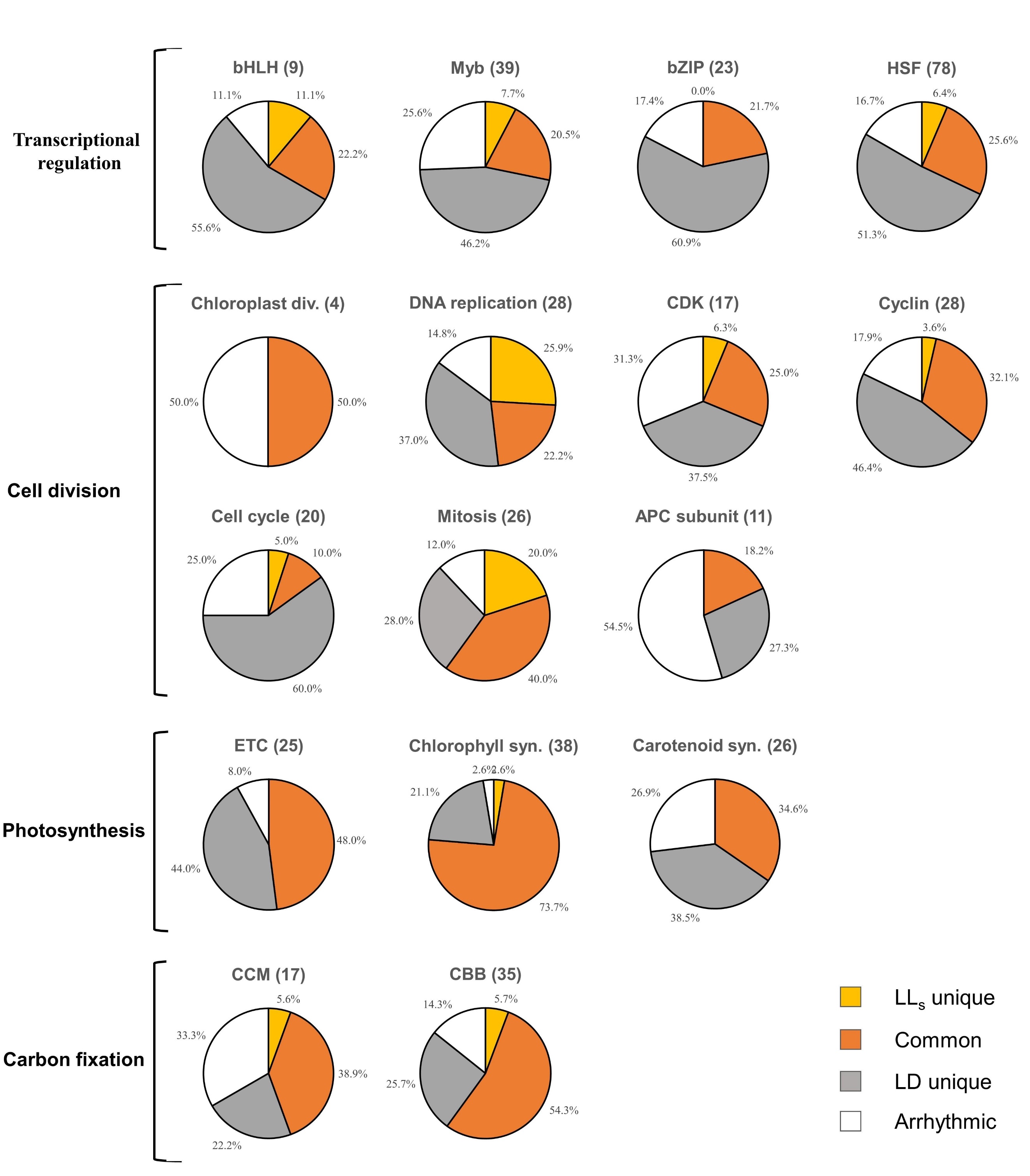


Fig. S6. Proportions of self-sustained rhythmic genes in selected biological processes in the short-term constant light (LL_s_) group compared with the light/dark cycle (LD) group. For a specific function or pathway, all annotated genes present in *P. tricornutum* were searched on PLAZA (https://bioinformatics.psb.ugent.be/plaza/versions/plaza_diatoms_01/). The pie charts indicate the proportions of LL_s_- or LD-derived rhythmic genes within each selected functional group. bHLH, basic-helix-loop-helix; Myb; bZIP, basic region leucine zipper; HSF, heat shock factor; Chloroplast div., chloroplast division; Chlorophyll syn., chlorophyll synthesis; Carotenoid syn., carotenoid synthesis; CCM, carbon concentrating mechanism; CBB, Calvin-Benson-Bassham cycle; CDK, cyclin-dependent kinase; APC, anaphase-promoting complex; ETC, electron transport chain.


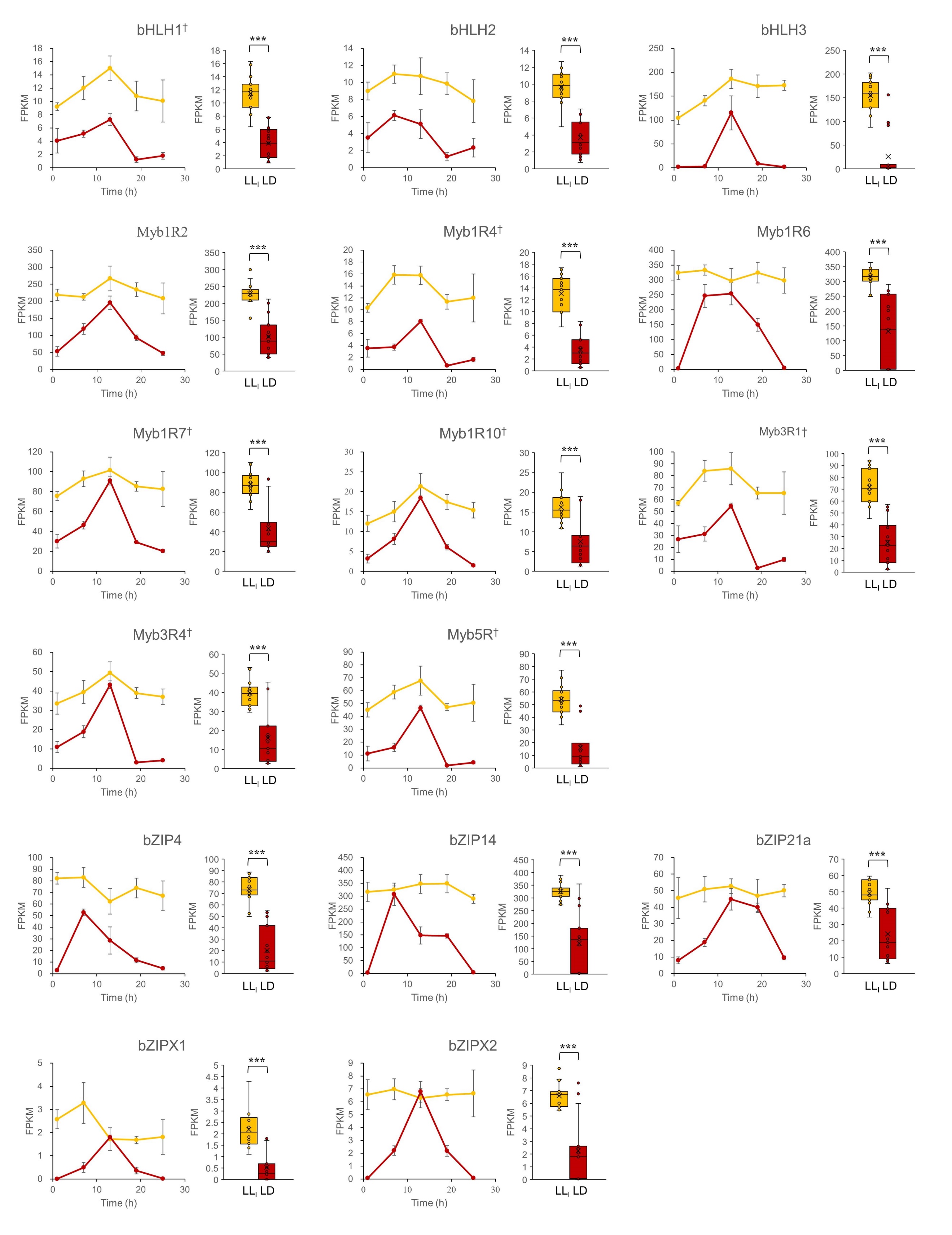


**Fig. S7.** Differentially expressed genes (DEGs) of transcription factors comparing expression levels under the long-term constant light (LL_l_) and light/dark cycle (LD) conditions. Differential expression analysis using all data across five sampling times covering 24 h was performed to compare gene expression levels between the LL_l_ and LD groups. DEGs with significant differences (P_adj_ < 0.05) and large fold changes (|log_2_foldchange| > 1) were screened. Expression data of each identified DEG include a trendline of average FPKM over the sampling period (24 h) and distribution statistics on the data across all time points; LL_l_ and LD are presented in yellow and red, respectively. (*, p-value < 0.05; **, p-value < 0.01; ***, p-value < 0.001. The label † indicates genes with significant rhythmicity under constant light conditions)


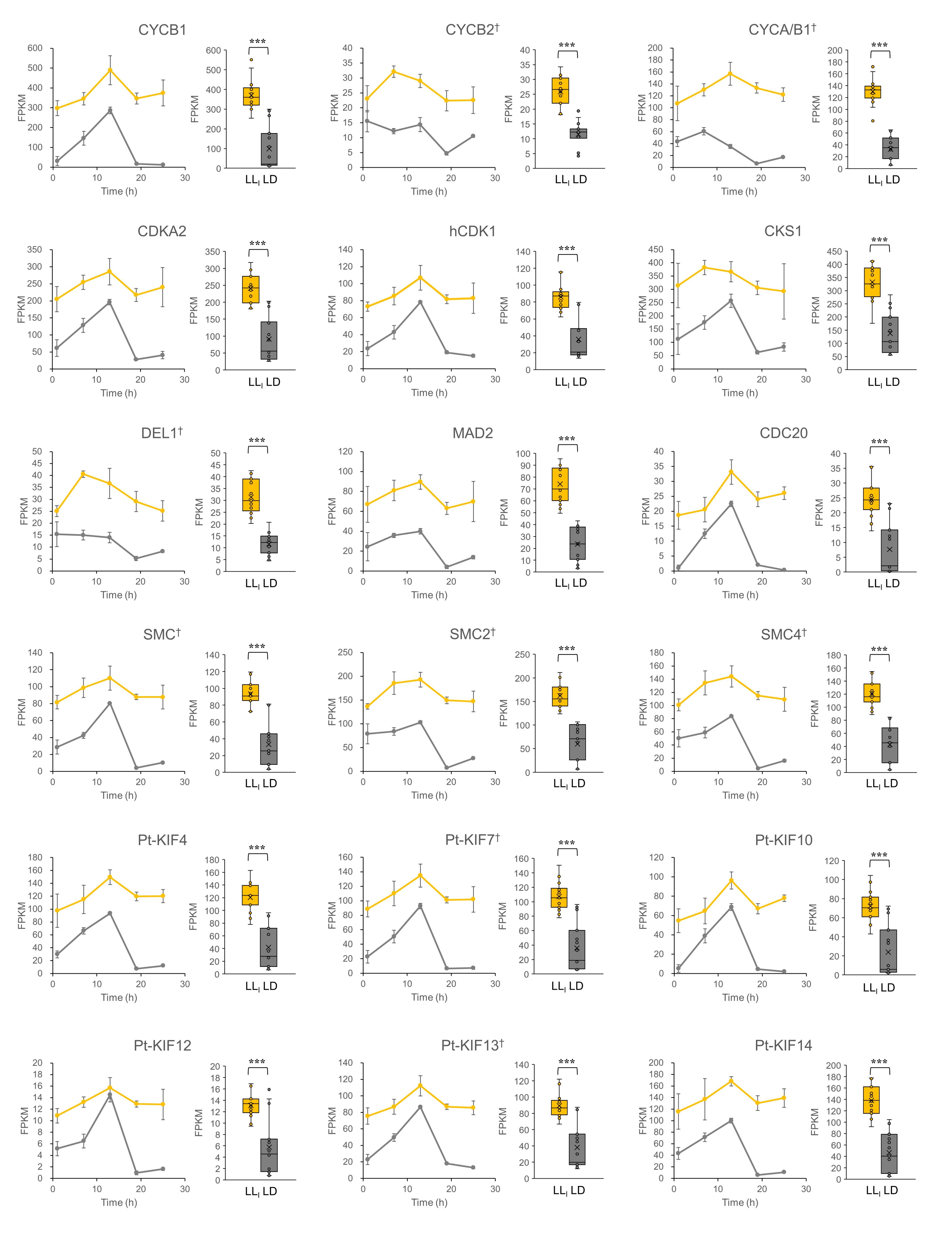


**Fig. S8.** Differentially expressed genes (DEGs) involved in cell division comparing expression levels under the long-term constant light (LL_l_) and light/dark cycle (LD) conditions. Differential expression analysis using all data across five sampling times covering 24 h was performed to compare gene expression levels between the LL_l_ and LD groups. DEGs with significant differences (P_adj_ < 0.05) and large fold changes (|log_2_foldchange| > 1) were screened. Expression data of each identified DEG include a trendline of average FPKM over the sampling period (24 h) and distribution statistics on the data across all time points; LL_l_ and LD are presented in yellow and grey, respectively. (*, p-value < 0.05; **, p-value < 0.01; ***, p-value < 0.001. The label † indicates genes with significant rhythmicity under constant light conditions).


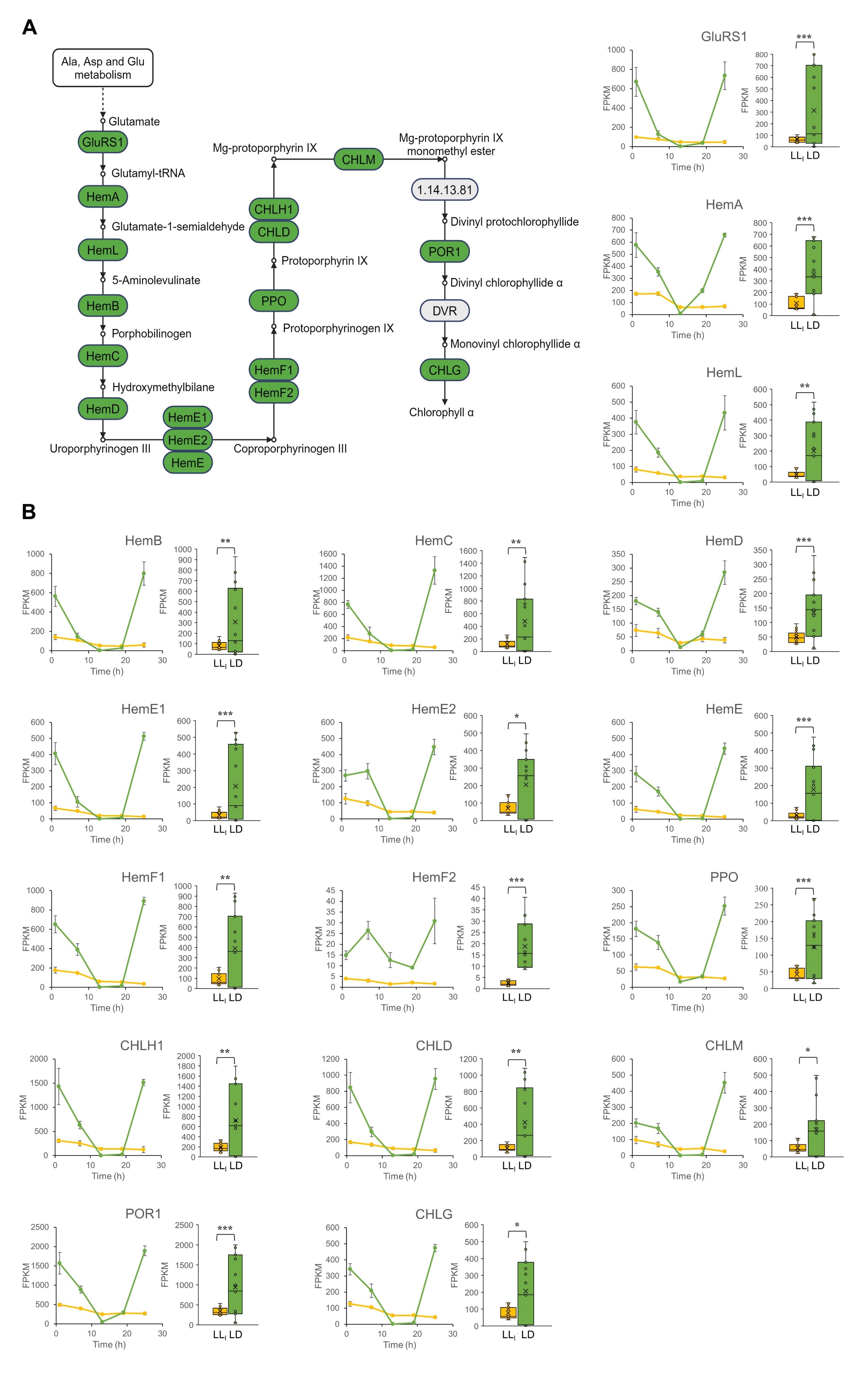


Fig. S9. Differentially expressed genes (DEGs) in chlorophyll biosynthesis comparing expression levels under the long-term constant light (LL_l_) and light/dark cycle (LD) conditions. Differential expression analysis using all data across five sampling times covering 24 h was performed to compare gene expression levels between the LL_l_ and LD groups. DEGs with significant differences (P_adj_ < 0.05) and large fold changes (|log_2_foldchange| > 1) were screened. *(A)* Mapping of DEGs (LL_l_/LD) in the metabolic pathways of chlorophyll biosynthesis, DEGs are highlighted in green color. *(B)* Expression data of each identified DEG, including a trendline of average FPKM over the sampling period (24 h) and distribution statistics on the data across all time points; LL_l_ and LD are presented in yellow and green, respectively. (*, p-value < 0.05; **, p-value < 0.01; ***, p-value < 0.001).


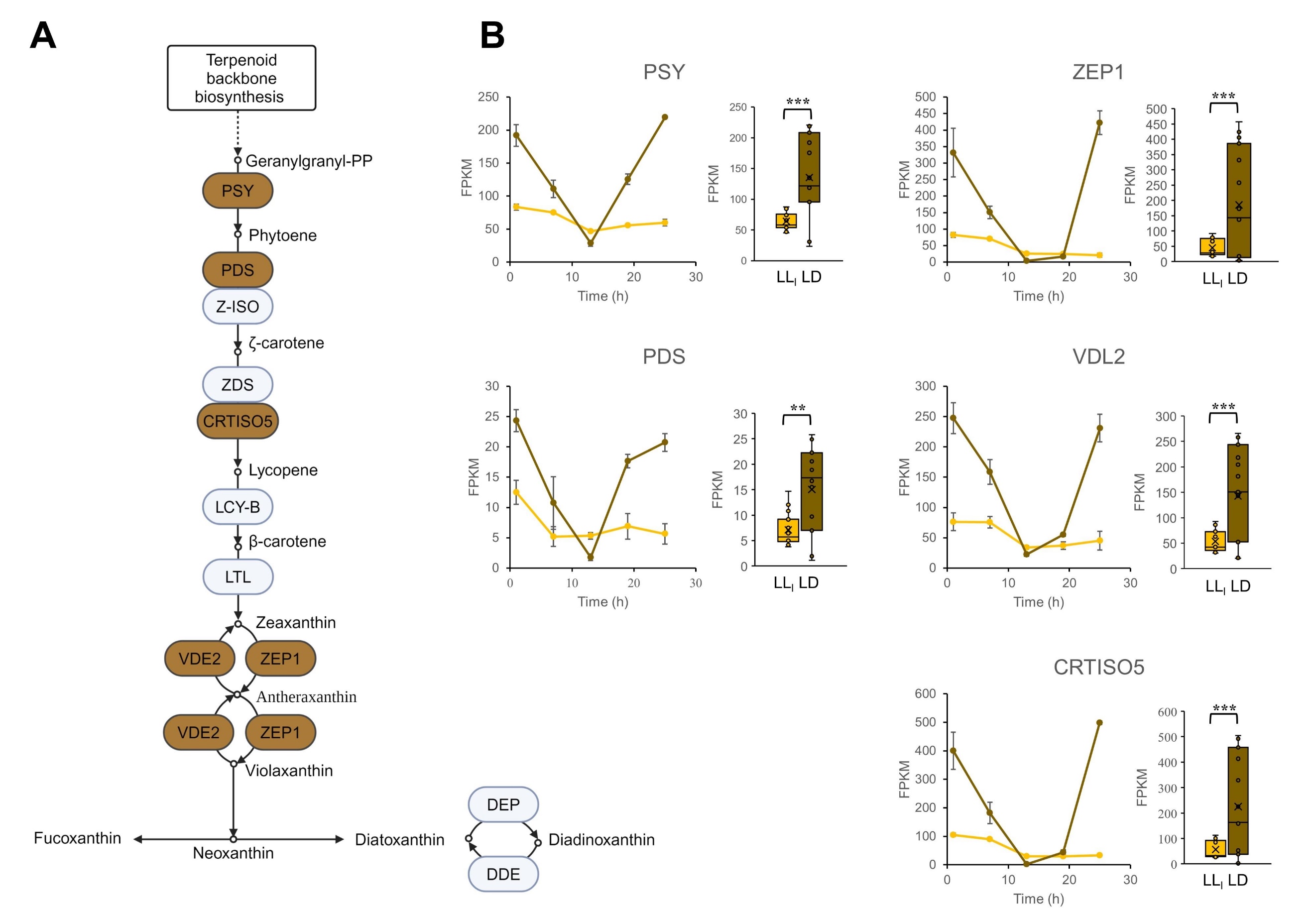


Fig. S10. Differentially expressed genes (DEGs) in carotenoid biosynthesis comparing expression levels under the long-term constant light (LL_l_) and light/dark cycle (LD) conditions. Differential expression analysis using all data across five sampling times covering 24 h was performed to compare gene expression levels between the LL_l_ and LD groups. DEGs with significant differences (P_adj_ < 0.05) and large fold changes (|log_2_foldchange| > 1) were screened. *(A)* Mapping of DEGs (LL_l_/LD) in the metabolic pathways of the carotenoid biosynthesis, DEGs are highlighted in brown color. *(B)* Expression data of each identified DEG, including a trendline of average FPKM over the sampling period (24 h) and distribution statistics on the data across all time points; LL_l_ and LD are presented in yellow and brown, respectively. (*, p-value < 0.05; **, p-value < 0.01; ***, p-value < 0.001)


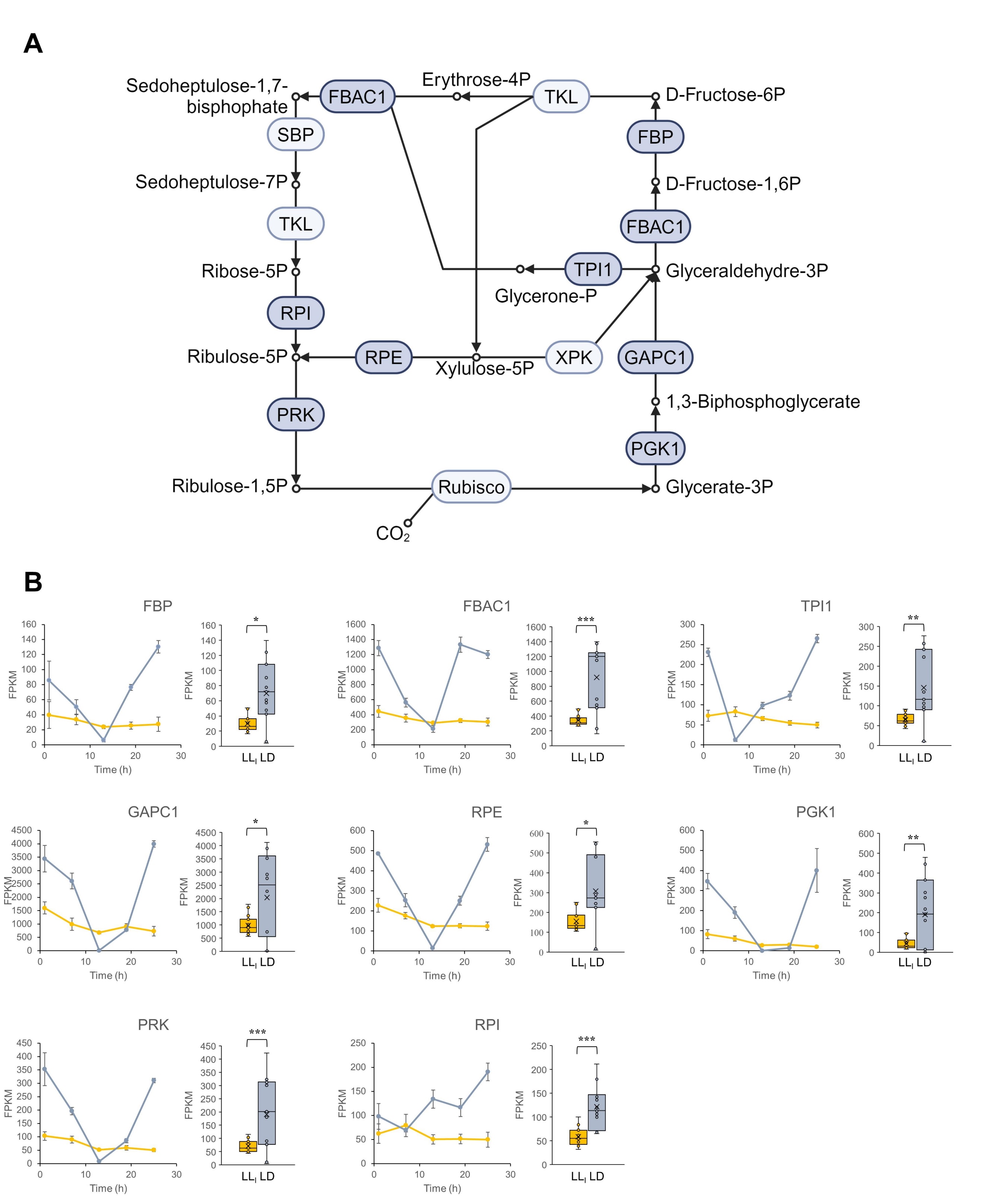


Fig. S11. Differentially expressed genes (DEGs) in the Calvin-Benson-Bassham (CBB) cycle comparing expression levels under the long-term constant light (LL_l_) and light/dark cycle (LD) conditions. Differential expression analysis using all data across five sampling times covering 24 h was performed to compare gene expression levels between the LL_l_ and LD groups. DEGs with significant differences (P_adj_ < 0.05) and large fold changes (|log_2_foldchange| > 1) were screened. *(A)* Mapping of DEGs (LL_l_/LD) in the metabolic pathways of the CBB cycle, DEGs are highlighted in blue color. *(B)* Expression data of each identified DEG, including a trendline of average FPKM over the sampling period (24 h) and distribution statistics on the data across all time points; LL_l_ and LD are presented in yellow and blue, respectively. (*, p-value < 0.05; **, p-value < 0.01; ***, p-value < 0.001).

Table S1. Growth performance of *P. tricornutum* under long-term constant light and light/dark cycle conditions (n = 3), respectively.

| Group | Semi-continuous cultivation period | | 24-h sampling period | |
| --- | --- | --- | --- | --- |
|  | μ (d^-1^) | P_b_ (mg L^-1^d^-1^) | μ (d^-1^) | P_b_ (mg L^-1^d^-1^) |
| LD | 0.51 $\pm$ 0.02 | 49.82 $\pm$ 0.77 | 0.46 $\pm$ 0.02 | 46.44 $\pm$ 1.62 |
| LL_l_ | 0.90 $\pm$ 0.19 | 79.08 $\pm$ 3.03 | 0.93 $\pm$ 0.18 | 81.43 $\pm$ 3.44 |

LL_l_, long-term constant light; LD, light/dark cycle; μ, growth rate; P_b_, biomass productivity.

Table S2. Rhythmic studies on microalgae at molecular levels.

| Algal species | Experimental conditions | | | Type of datasets | Rhythmic detection algorithm | References |
| --- | --- | --- | --- | --- | --- | --- |
|  | Light/dark cycle condition | Constant light conditions immediately after light/dark cycles | Long-term constant light conditions |  |  |  |
| *Phaeodactylum tricornutum* | + | + | + | RNAseq | JTK | This study |
| *Phaeodactylum tricornutum* | - | + | - | qPCR | NA | (17) |
| *Skeletonema costatum* | - | + | - | qPCR | NA | (17) |
| *Phaeodactylum tricornutum* | - | + | - | qPCR | FFT-NLLS | (18) |
| *Phaeodactylum tricornutum* | + | - | - | qPCR | NA | (19) |
| *Seminavis robusta* | + | - | - | RNAseq | empJTK | (20) |
| *Thalassiosira pseudonana* | + | - | - | RNAseq | NA | (21) |
| *Chlamydomonas reinhardtii* | + | - | - | RNAseq | JTK | (22) |
| *Cyanophora paradoxa* | + | - | - | RNAseq | JTK | (22) |
| *Porphyridium purpureum* | + | - | - | RNAseq | JTK | (22) |
| *Phaeodactylum tricornutum* | + | - | - | RNAseq | NA | (23) |
| *Nannochloropsis oceanica* | + | - | - | RNAseq | COSPOT, DFT | (24) |
| *Chlamydomonas reinhardtii* | + | - | - | RNAseq | JTK | (25) |
| *Thalassiosira pseudonana* | + | - | - | microarray | Limma | (26) |
| *Phaeodactylum tricornutum* | + | - | - | microarray | Limma | (27) |
| *Ostreococcus tauri* | + | - | - | microarray | Bayesiian Fourier clustering | (28) |
| *Chlamydomonas reinhardtii* | - | + | - | microarray | Cosiner | (29) |

LD, light/dark cycle; LL, constant light; JTK, Jonckheere-Terpstra-Kendall (30); empJTK, empirical JTK (31); FFT-NLLS, Fast Fourier nonlinear least square (32); COSPOT, Circadian Oscillation in Single-cell Profiles Over Time (33); DFT, discrete Fourier transform (34); NA, not available.

Table S3. Potential mRNA targets of differentially expressed miRNAs under the long-term constant light condition in comparison with the light/dark cycle condition.

| Differentially expressed miRNAs | Total number of miRNA | Total number of target mRNA | Target mRNAs of interest | |
| --- | --- | --- | --- | --- |
|  |  |  | Up-regulated | Down-regulated |
| Up-regulated miRNA | 18 | 751 | 33 | 40 |
| Down-regulated miRNA | 64 | 1281 | 47 | 63 |

Dataset S1 (separate file). Physiological data in semi-continuous cultivation.

Dataset S2 (separate file). Statistics of genome mapping of coding RNAs.

Dataset S3 (separate file). Statistics of genome mapping and annotation for small RNAs.

Dataset S4 (separate file). Expression data of mRNA and miRNA.

Dataset S5 (separate file). Rhythmic detection for mRNAs and miRNAs.

Dataset S6 (separate file). Differential expression analysis on mRNAs and miRNAs.

Dataset S7 (separate file). Rhythmic genes and differentially expressed genes in selected biological processes.

Dataset S8 (separate file). Targets of differentially expressed microRNAs.

**SI References**

1. A. M. Bolger, M. Lohse, B. Usadel, Trimmomatic: a flexible trimmer for Illumina sequence data. *Bioinformatics* **30**, 2114-2120 (2014).

2. D. Kim, B. Langmead, S. L. Salzberg, HISAT: a fast spliced aligner with low memory requirements. *J Nature Methods* **12**, 357-360 (2015).

3. M. Pertea *et al.*, StringTie enables improved reconstruction of a transcriptome from RNA-seq reads. *J Nature Biotechnology* **33**, 290-295 (2015).

4. S. Anders, P. T. Pyl, W. Huber, HTSeq-a Python framework to work with high-throughput sequencing data. *Bioinformatics* **31**, 166-169 (2015).

5. A. Roberts, H. Pimentel, C. Trapnell, L. Pachter, Identification of novel transcripts in annotated genomes using RNA-Seq. *Bioinformatics* **27**, 2325-2329 (2011).

6. B. Langmead, Aligning short sequencing reads with Bowtie. *Current Protocols in Bioinformatics* **32**, 11.17. 11-11.17. 14 (2010).

7. S. F. Altschul, W. Gish, W. Miller, E. W. Myers, D. J. Lipman, Basic local alignment search tool. *Journal of Molecular Biology* **215**, 403-410 (1990).

8. S. Griffiths-Jones, A. Bateman, M. Marshall, A. Khanna, S. R. Eddy, Rfam: an RNA family database. *Nucleic Acids Research* **31**, 439-441 (2003).

9. S. Griffiths-Jones, H. K. Saini, S. Van Dongen, A. J. Enright, miRBase: tools for microRNA genomics. *Nucleic Acids Research* **36**, D154-D158 (2007).

10. M. R. Friedländer, S. D. Mackowiak, N. Li, W. Chen, N. Rajewsky, miRDeep2 accurately identifies known and hundreds of novel microRNA genes in seven animal clades. *Nucleic Acids Research* **40**, 37-52 (2012).

11. M. I. Love, W. Huber, S. Anders, Moderated estimation of fold change and dispersion for RNA-seq data with DESeq2. *Genome Biology* **15**, 1-21 (2014).

12. A. Enright *et al.*, MicroRNA targets in Drosophila. *Genome Biology* **4**, 1-27 (2003).

13. A. Huang, L. He, G. Wang, Identification and characterization of microRNAs from Phaeodactylum tricornutum by high-throughput sequencing and bioinformatics analysis. *BMC Genomics* **12**, 1-11 (2011).

14. N. Tuteja, N. Q. Tran, H. Q. Dang, R. Tuteja, Plant MCM proteins: role in DNA replication and beyond. *Plant Molecular Biology* **77**, 537-545 (2011).

15. P. Bulankova, G. Bilcke, W. Vyverman, L. De Veylder, "Cellular Hallmarks and Regulation of the Diatom Cell Cycle" in The Molecular Life of Diatoms*,* A. Falciatore, T. Mock, Eds. (Springer International Publishing, Cham, 2022), 10.1007/978-3-030-92499-7_9, pp. 229-263.

16. J. Levering, C. L. Dupont, A. E. Allen, B. O. Palsson, K. Zengler, Integrated regulatory and metabolic networks of the marine diatom Phaeodactylum tricornutum predict the response to rising CO2 levels. *Msystems* **2**, e00142-00116 (2017).

17. S. Zhang, Y. Wu, L. Lin, D. Wang, Molecular insights into the circadian clock in marine diatoms. *Acta Oceanologica Sinica* **41**, 87-98 (2022).

18. R. Annunziata *et al.*, bHLH-PAS protein RITMO1 regulates diel biological rhythms in the marine diatom Phaeodactylum tricornutum. *PNAS* **116**, 13137-13142 (2019).

19. G. E. Kayanja, I. M. Ibrahim, S. Puthiyaveetil, Regulation of Phaeodactylum plastid gene transcription by redox, light, and circadian signals. *Photosynthesis Research* **147**, 317-328 (2021).

20. G. Bilcke *et al.*, Diurnal transcript profiling of the diatom Seminavis robusta reveals adaptations to a benthic lifestyle. *Plant J* **107**, 315-336 (2021).

21. J. A. Goldman *et al.*, Fe limitation decreases transcriptional regulation over the diel cycle in the model diatom Thalassiosira pseudonana. *J PLoS One* **14**, e0222325 (2019).

22. C. Ferrari *et al.*, Kingdom-wide comparison reveals the evolution of diurnal gene expression in Archaeplastida. *J Nature Communications* **10**, 737 (2019).

23. S. R. Smith *et al.*, Transcriptional Orchestration of the Global Cellular Response of a Model Pennate Diatom to Diel Light Cycling under Iron Limitation. *PLoS Genet* **12**, e1006490 (2016).

24. E. Poliner *et al.*, Transcriptional coordination of physiological responses in N annochloropsis oceanica CCMP 1779 under light/dark cycles. *J The Plant Journal* **83**, 1097-1113 (2015).

25. J. M. Zones, I. K. Blaby, S. S. Merchant, J. G. Umen, High-Resolution Profiling of a Synchronized Diurnal Transcriptome from Chlamydomonas reinhardtii Reveals Continuous Cell and Metabolic Differentiation. *Plant Cell* **27**, 2743-2769 (2015).

26. J. Ashworth *et al.*, Genome-wide diel growth state transitions in the diatom Thalassiosira pseudonana. *J Proceedings of the National Academy of Sciences* **110**, 7518-7523 (2013).

27. M. S. Chauton, P. Winge, T. Brembu, O. Vadstein, A. M. Bones, Gene regulation of carbon fixation, storage, and utilization in the diatom Phaeodactylum tricornutum acclimated to light/dark cycles. *Plant Physiol* **161**, 1034-1048 (2013).

28. A. Monnier *et al.*, Orchestrated transcription of biological processes in the marine picoeukaryote Ostreococcus exposed to light/dark cycles. *BMC Genomics* **11**, 192 (2010).

29. K.-i. Kucho, K. Okamoto, S. Tabata, H. Fukuzawa, M. Ishiura, Identification of novel clock-controlled genes by cDNA macroarray analysis in Chlamydomonas reinhardtii. *Plant Molecular Biology* **57**, 889-906 (2005).

30. M. E. Hughes, J. B. Hogenesch, K. Kornacker, JTK_CYCLE: an efficient nonparametric algorithm for detecting rhythmic components in genome-scale data sets. *Journal of biological rhythms* **25**, 372-380 (2010).

31. A. L. Hutchison, R. Allada, A. R. Dinner, Bootstrapping and empirical Bayes methods improve rhythm detection in sparsely sampled data. *Journal of biological rhythms* **33**, 339-349 (2018).

32. T. Zielinski, A. M. Moore, E. Troup, K. J. Halliday, A. J. Millar, Strengths and limitations of period estimation methods for circadian data. *PloS one* **9**, e96462 (2014).

33. S. Panda *et al.*, Coordinated transcription of key pathways in the mouse by the circadian clock. *Cell* **109**, 307-320 (2002).

34. N. Panchy *et al.*, Prevalence, evolution, and cis-regulation of diel transcription in Chlamydomonas reinhardtii. *G3: Genes, Genomes, Genetics* **4**, 2461-2471 (2014).
